# Circulating small RNA signatures differentiate accurately the subtypes of muscular dystrophies: small-RNA next-generation sequencing analytics and functional insights

**DOI:** 10.1080/15476286.2022.2058817

**Published:** 2022-04-07

**Authors:** Andrea C. Kakouri, Demetris Koutalianos, Andrie Koutsoulidou, Anastasis Oulas, Marios Tomazou, Nikoletta Nikolenko, Chris Turner, Andreas Roos, Anna Lusakowska, Katarzyna Janiszewska, George K. Papadimas, Constantinos Papadopoulos, Evangelia Kararizou, Eleni Zamba Papanicolaou, Grainne Gorman, Hanns Lochmüller, George M. Spyrou, Leonidas A. Phylactou

**Affiliations:** a Department of Bioinformatics, The Cyprus Institute of Neurology and Genetics, Nicosia, Cyprus; b Department of Molecular Genetics, Function & Therapy, The Cyprus Institute of Neurology and Genetics, Nicosia, Cyprus; c Department of Neurogenetics, The Cyprus Institute of Neurology and Genetics, Nicosia, Cyprus; d National Hospital for Neurology and Neurosurgery, Queen Square, University College London Hospitals NHS Foundation Trust, London, UK; e Department of Neuropediatrics, University Hospital Essen, Duisburg-Essen University, Germany; f Division of Neurology, Department of Medicine, Childrens Hospital of Eastern Ontario Research Institute, University of Ottawa, Ottawa, ON, Canada; g Department of Neurology, Medical University of Warsaw, Warsaw, Poland; h Department of Neurology, Central Hospital of Medical University of Warsaw, Poland; i Department of Neurology, Eginitio hospital, Medical School of Athens, Athens, Greece; j Neuroepidemiology Department, Cyprus Institute of Neurology & Genetics, Nicosia, Cyprus; k Wellcome Trust Centre for Mitochondrial Research, Institute of Neuroscience, University of Newcastle, Newcastle, UK; l Division of Neurology, Department of Medicine, The Ottawa Hospital, Ottawa, ON, Canada; m Centro Nacional de AnálisisGenómico, Center for Genomic Regulation (CNAG-CRG), Barcelona Institute of Science and Technology (Bist), Barcelona, Spain

**Keywords:** Small RNAs, miRNAs, NGS, biomarkers, muscular dystrophies

## Abstract

Muscular dystrophies are a group of rare and severe inherited disorders mainly affecting the muscle tissue. Duchene Muscular Dystrophy, Myotonic Dystrophy types 1 and 2, Limb Girdle Muscular Dystrophy and Facioscapulohumeral Muscular Dystrophy are some of the members of this family of disorders. In addition to the current diagnostic tools, there is an increasing interest for the development of novel non-invasive biomarkers for the diagnosis and monitoring of these diseases. miRNAs are small RNA molecules characterized by high stability in blood thus making them ideal biomarker candidates for various diseases. In this study, we present the first genome-wide next-generation small RNA sequencing in serum samples of five different types of muscular dystrophy patients and healthy individuals. We identified many small RNAs including miRNAs, lncRNAs, tRNAs, snoRNAs and snRNAs, that differentially discriminate the muscular dystrophy patients from the healthy individuals. Further analysis of the identified miRNAs showed that some miRNAs can distinguish the muscular dystrophy patients from controls and other miRNAs are specific to the type of muscular dystrophy. Bioinformatics analysis of the target genes for the most significant miRNAs and the biological role of these genes revealed different pathways that the dysregulated miRNAs are involved in each type of muscular dystrophy investigated. In conclusion, this study shows unique signatures of small RNAs circulating in five types of muscular dystrophy patients and provides a useful resource for future studies for the development of miRNA biomarkers in muscular dystrophies and for their involvement in the pathogenesis of the disorders.

## Introduction

Muscular dystrophies are a group of rare inherited disorders characterized by muscle wasting and weakness of variable distribution and severity. They are characterized by a variety of clinical phenotypes, severity, age of onset and rate of progression. Furthermore, in several muscular dystrophies additional tissues and organs are affected such as the heart and the central nervous system (CNS) [1,2]. Some of the most common forms of muscular dystrophies are the Duchene Muscular Dystrophy (DMD), the Myotonic Dystrophy type 1 and 2 (DM1 and DM2), the Limb-Girdle Muscular Dystrophy (LGMD) and the Facioscapulohumeral Muscular Dystrophy (FSHD).

DMD is the most common and severe inherited muscular dystrophy of childhood [3]. It is caused by mutations in the X-linked *dystrophin* gene abolishing the expression of the dystrophin protein [3]. DMD is clinically characterized by progressive muscle necrosis and wasting leading to loss of ambulation by 8–12 years of age and death by early adulthood due to cardiorespiratory failure [4]. DM1 is the second most common type of muscular dystrophy after DMD and the most common type of muscular dystrophy in adults [5]. It is an inherited autosomal dominant, neuromuscular disorder caused by a trinucleotide CTG repeat expansion in the 3′UTR of the *dystrophia myotonica protein kinase* (*DMPK*) gene located on the chromosome 19 [6]. DM1 initially affects the skeletal muscles through progressive skeletal muscle weakness, wasting and myotonia however, it is considered as a multi-systemic disorder since it affects other tissues such as the heart and the CNS [7]. DM2 is a second type of myotonic dystrophy and is inherited in an autosomal dominant pattern like DM1. It is caused by a pathogenic expansion of a CCTG tetranucleotide expansion in the *CCHC-type zinc finger nucleic acid-binding protein* (*CNBP)* gene [8]. In DM2, muscles and other tissues such as the heart, the eyes and the pancreas are affected. It is characterized by extended muscle tensing (myotonia) as well as muscle weakness, stiffness and pain [8]. Another rare autosomal dominant inherited muscular disorder is FSHD. FSHD is a progressive disorder that has been classified into two types, FSHD1 and FSHD2. Both forms of FSHD display identical clinical phenotype but different genetic and epigenetic basis. FSHD1 which is the most common form of the FSHD, is caused by a deletion of a key number of repetitive elements on chromosome 4q35 [9–11]. FSHD2, counts only the 5% of the FSHD patients and has been linked to mutations in the *structural maintenance of chromosomes flexible hinge domain containing 1* (*SMCHD1*) gene on chromosome 18. Both forms of FSHD are characterized by progressive weakness and atrophy of the skeletal muscles of the face, shoulder, arm and abdominal muscles as well as in other areas of the body [12–17]. LGMD is a diverse group of severe muscular dystrophies which affects the voluntary muscles around the hips and shoulders. There are many LGMD subtypes categorized by the gene causing the disease and the type of inheritance. The LGMD R1 calpain3-related is the most prevalent form of LGMD cases and is caused by mutations in the *Calpain 3 (CAPN3)* gene encoding for a neutral protease [18]. The progression of these diseases leads to the loss of muscle strength and bulk over a number of years [19]. While different genetic defects cause the five distinct types of muscular dystrophy included in our study, there are some notable similarities of muscular pathology and downstream molecular pathways between them, which may have an impact on miRNA signatures. For example, secondary inflammatory changes are observed in both DMD and FSHD [14,20]. Furthermore, differences in muscle fibre sizes exist between the five muscular dystrophies under investigation. In particular, muscle fibre hypertrophy is observed in all the five types of muscular dystrophy, whereas muscle atrophy is observed only in DM1, DM2 and DMD [14,20–22]. Additionally, internal nuclei are present in muscle pathologies of all the five muscular dystrophies, although in different extent [21–24].

Genetic tests are currently being used for the diagnosis of muscular dystrophies and physical examinations by the clinicians for monitoring the progress of the disease. An additional diagnostic tool which could complement and even replace the existing diagnostic and monitoring methods will help clinicians to have a greater understanding of their patients’ progress. Currently, there is an increasing interest for the discovery of biomarkers for this family of diseases. Although strong emphasis has been placed on the research for identifying biomarkers for DMD, limited work has been performed regarding the identification of biomarkers for other muscular dystrophies such as DM1, DM2, LGMD and FSHD [25–27]. Nowadays, there is an intense interest for the discovery of new and more accurate biomarkers. More specifically, the potential use of miRNAs as biomarkers has been highlighted in literature for numerous diseases and conditions. miRNAs are small non-coding RNA molecules that control a numerous of biological processes [28–31]. They have been found to stably circulate in blood and were suggested as ideal diagnostic, prognostic, monitoring or pharmacodynamic candidate biomarkers for various diseases and conditions including neuromuscular disorders [32–39].

Currently, the majority of research regarding the development of miRNAs as biomarkers for muscular dystrophies is targeted to specific miRNAs for each type of muscular dystrophy under investigation [40–45]. Additionally, there are some reports that miRNA arrays were used for the screening of miRNAs in plasma or serum of muscular dystrophies [46]. Four miRNAs specifically expressed in muscle tissue, miR-1, miR-133a, miR-133b and miR-206, known as myomiRs, were reported to be elevated in blood of DMD patients and DMD animal models compared to controls [43,47–53]. Importantly, the levels of these miRNAs were correlated with the disease severity and clinical assessments of the patients [47,50,54]. Additionally, miR-483 was found to be increased in blood of DMD patients and DMD animal model compared to controls [55]. The four myomiRs identified as biomarkers for DMD, miR-1, miR-133a, miR-133b and miR-206, were also reported to be elevated in DM1 patients compared to healthy individuals [42,56,57]. Notably, the levels of these miRNAs were correlated with the progression of the disease [42,56]. Additional miRNAs were determined as biomarkers for DM1 and some of these were also reported as biomarkers for DM2 [46,57]. The identification of common miRNA candidate biomarkers for different types of muscular dystrophies implies that their release in the blood of the patients is a consequence of muscle degradation that is a common characteristic for all the muscular dystrophies and not as a result of the specific disease cause.

Although several attempts have been made to identify miRNA biomarkers for muscular dystrophies, a larger and deeper screening is needed to profile all the small RNAs that circulate in the blood of muscular dystrophy patients, in order to develop reliable biomarkers. The discovery of miRNAs or other small RNAs that are differentially expressed in the blood circulation of muscular dystrophy patients would provide evidence for identifying the best candidate biomarkers that can help to distinguish the patients of muscular dystrophies from the healthy people and also to discriminate the patients from each type of muscular dystrophy.

In this work, we aimed to provide an in-depth analysis of the small RNA molecules that circulate in the blood of muscular dystrophy patients and suggest reliable miRNA candidate biomarkers for these disorders. We performed the first genome-wide comparative small RNA analysis in serum samples of patients with five different types of muscular dystrophies, DMD, DM1, DM2, FSHD1 and LGMD R1 calpain3-related, and healthy individuals, using a conventional method of data analysis, as well as a machine learning approach. We identified the differentially expressed small RNAs such as miRNAs, long-non-coding RNAs (lncRNAs), transfer RNAs (tRNAs), small nucleolar RNAs (snoRNAs) and small nuclear RNAs (snRNA), that can distinguish the muscular dystrophy patients from healthy people and also the specific type of muscular dystrophy that the patients are affected. Here we show a number of differentially expressed miRNAs, some of which were previously associated with muscular dystrophies, as well as previously uncharacterized miRNAs that could clearly separate muscular dystrophy patients from healthy individuals. Further bioinformatics analysis of the target genes of the most significant miRNAs and the biological role of the target genes discovered distinct pathways that the dysregulated miRNAs are involved in each type of muscular dystrophy under investigation. In conclusion, in this study we show that each type of muscular dystrophy has a unique signature of small RNA molecules circulating in the blood of patients and we provide novel evidence regarding the miRNA candidate biomarkers in muscular dystrophies and their role in the pathogenesis of the disorders.

## Results

### Identification of small RNAs in serum of muscular dystrophies patients

**Figure 1.**
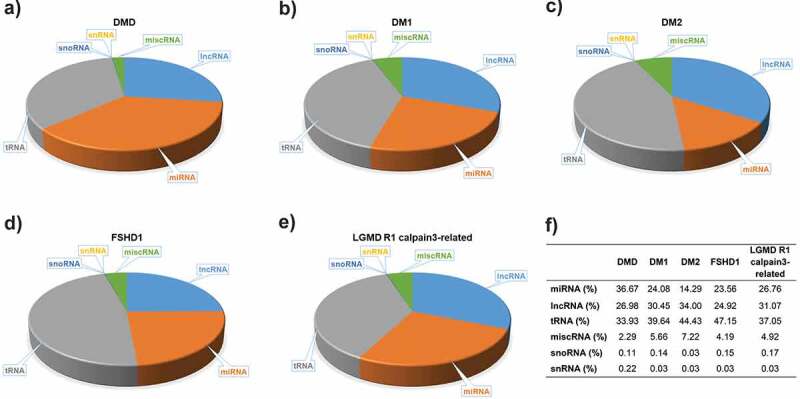
**Small RNA molecules in serum of five muscular dystrophies**. A substantial proportion of the circulating serum small RNAs content from each of the muscular dystrophies, A) DMD, B) DM1, C) DM2, D) FSHD1 and E) LGMD R1 calpain3-related, was determined. F) The percentages of distinct small RNA molecules population in each type of muscular dystrophy.

Serum samples from eight patients of each of the five muscular dystrophies, DMD, DM1, DM2, FSHD1 and LGMD R1 calpain3-related and, as well twenty-two age- and gender-matched healthy participants were used for the screening of the total small RNAs (Table S1). DNA libraries were created from the RNA samples isolated from the serum samples. Small RNA Next-Generation Sequencing (NGS) was next performed for all the samples to profile the entire spectrum of small RNA molecules present in the serum of the patients and the healthy participants. The average total reads revealed from all the NGS experiments were ranged between 5,451,364 and 9,869,822. Small RNA NGS revealed a distinct population of different circulating small RNA molecules for each type of muscular dystrophy. The largest portion of small RNA molecules was mapped as miRNAs, lncRNAs and tRNAs for all the five types of muscular dystrophies under investigation however distinct small RNA molecules population was observed in the different types of muscular dystrophy (Fig. 1).

### Identification of differentially expressed miRNAs

**Figure 2.**
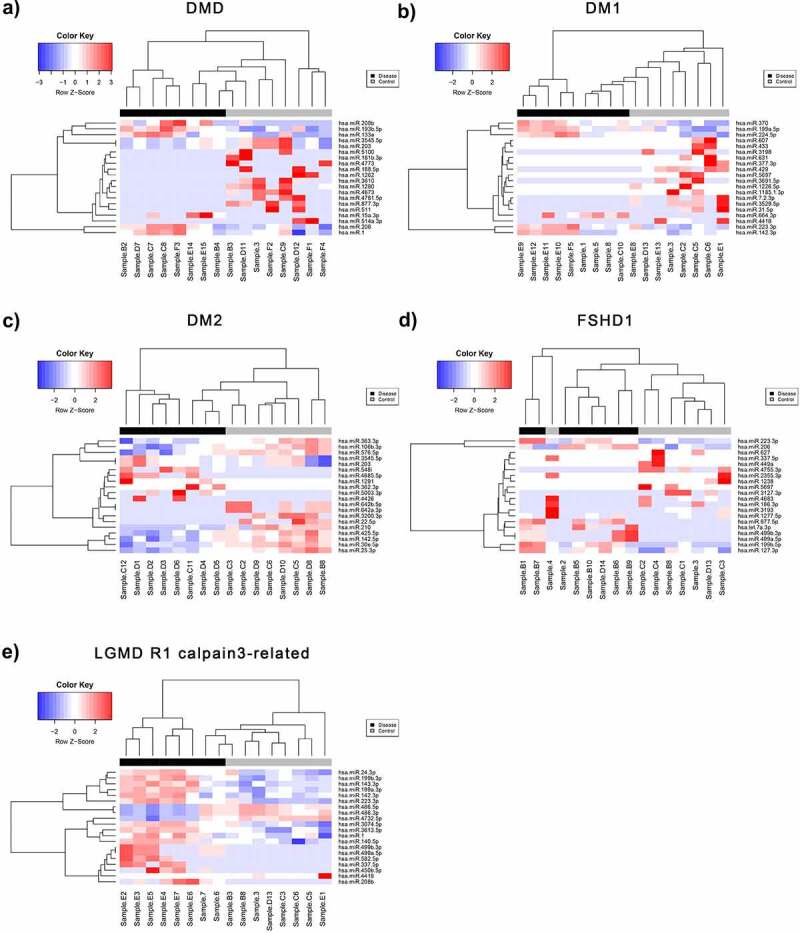
**Differentially expressed miRNAs (DEmiRNAs) between control and patient of each muscular dystrophies**. Heatmaps of the top DEmiRNAs based on p-value for each of the five diseases A) DMD, B) DM1, C) DM2, D) FSHD1 and E) LGMD R1 calpain3-related. The controls group (healthy individuals) is shown in grey colour and the muscular dystrophy patients’ group is shown in black colour. The colour key panel shows the Z-score values calculated for each miRNA, by subtracting the row-mean and then dividing by the standard deviation. Z-scores describe the expression of each miRNA in relation to the mean. Overexpressed miRNAs are shown in red, under-expressed miRNAs in blue. White colour indicates expression change close to 0. Hierarchical clustering was performed for samples and miRNAs.

Following the pre-processing of raw data obtained from the small RNA NGS runs, the normalized count matrices were used to identify differentially expressed miRNAs between control and patient datasets for each of the five diseases of interest (DM1, DM2, DMD, FSHD1 and LGMD R1 calpain3-related). A total of 948 differentially expressed miRNAs (DEmiRNAs) were identified for DM1, of which 175 (18.5%) were significant (p-value < 0.05 and absolute log_2_FC > 1). For DM2, 1014 DEmiRNAs were calculated and 179 (17.7%) had p-value < 0.05 and absolute log_2_FC > 1. A total of 819 miRNAs were differentially expressed in DMD patient samples compared to controls, of which 100 (12.2%) were significant under the described conditions. For LGMD R1 calpain3-related, 152 out of 915 miRNAs were significantly differentially expressed (16.6%), while for FSHD 931 DEmiRNAs were calculated, of which 126 (13.5) were significant (p-value < 0.05 and absolute log_2_FC >1) (Figure S1). In all the sets, we have identified both over- and under-expressed miRNAs in patients with log_2_FC values ranging from ~ −6.5 to +5.5 and p-values <0.05. After adjusting for multiple testing, the miRNAs with a False Discover Rate (FDR) <0.05 were determined and shown in bold in each table (Tables S2-S6). Patients with LGMD R1 calpain3-related (Table S6) were identified with the highest number of statistically significant differentially expressed miRNAs (>20) following the FDR correction while DM2 and FSHD1 with the lowest – 3 miRNAs each. Volcano plots of statistical significance (-log10 (p-value) versus log_2_ fold change) were produced for each disease: DMD, DM1, DM2, FSHD1 and LGMD R1 calpain3-related (Figure S1), in order to summarize the results of differential expression analysis. The miRNAs with significant under- and over-expression (p-value < 0.05 and absolute log_2_FC > 1) are shown in blue and red colour, respectively, whereas the miRNAs with p-value > 0.05 or absolute log_2_FC < 1 are designated in black colour. An average of 76 under- and 70 over-expressed miRNAs were found for the five diseases. Principal Component Analysis (PCA) plots were also generated to evaluate the separation of patient and control samples based on their miRNA expression profiles (Figure S2). The two groups were clearly distinguished in most cases, with an average percentage variance of greater than 75% (Table S7). The separation of muscular dystrophy patients from healthy individuals is also shown in the heatmaps of Fig. 2, where the top DEmiRNAs based on p-value are presented and hierarchically clustered between the samples.

### Machine learning data analysis for detection of optimal set of miRNA gene panels

A machine learning approach was utilized to obtain a significant set of miRNA gene panels that act synergistically to optimally classify disease and control samples for all the five muscular dystrophies (DMD, DM1, DM2, FSHD1 and LGMD R1 calpain3-related). Individual p-values (although reported) are not as important in this approach, as is the cumulative effect of the panel of miRNAs acting in synergy. Therefore, some differences exist between the importance and p-values obtained from the machine learning approach compared with the differential expression analysis performed using edgeR.

Initially, the samples were separated into two classes; disease and control, in order to train the Support Vector Machine (SVM) classifier derived from the R e1071 library [58]. A leave-one-out cross-validation (LOOCV) procedure was used to assess the classifier performance. During each round of cross validation, each sample was removed recursively from the dataset and feature selection was performed on the remaining samples in the dataset. The model was then trained and utilized to classify the left-out sample. For the feature selection (miRNA selection), edgeR was used as previously described [59]. To find the optimal set of miRNA genes, the leave-one-out cross-validation method was performed by testing initially the top six miRNAs (with lowest p-value) and sequentially increasing the number of miRNAs for each run until classification accuracy reached a saturation point with no further improvement. The flow chart of the classification procedure is analysed in detail in the Material and Methods. The optimum set was different for each of the disease-control pairs assessed. Top miRNAs were selected for downstream target prediction analysis as well as network and pathway analysis. To assess the performance of each feature selection run, the accuracy, specificity, sensitivity and Matthew’s correlation coefficient (MCC) were calculated. MCC is defined as a balanced measurement of the classification quality which considers true and false positives and negatives. MCC return values within the range of [−1, 1]. PredictABEL [60] functions were used to assess model performance, including: the *plotRoc* function for plotting receiver operating characteristic (ROC) curves and calculating area under the curve (AUC) values. PredictABEL also includes functions for graphical representation of statistical results such as: distributions (*plotRiskDistribution* function), discrimination box plot (*plotDiscriminationBox* function) (Table 1; Supplementary Figures S3-7). Full lists of the selected pooled number and IDs of top miRNAs selected for each analysed disease-pair, are shown in supplementary information Tables S8-S12.

**Table 1.** ROC analysis for the polled miRNAs for each of the five muscular dystrophies

	Optimal set of LOOCV miRNAs	Pooled unique miRNAs	AUC
DM1	8	23	0.875
DM2	16	44	0.969
DMD	8	8	0.797
FSHD1	26	69	0.875
LGMD R1 calpain3-related	6	12	0.859

### Gene target prediction and functional analysis of the differentially expressed miRNA sets

The functional role of the identified DEmiRNAs was investigated further through gene target prediction. Probing the available miRNA databases in CluePedia Cytoscape plugin, we obtained sets of potential gene targets for the miRNA sets for each disease. Genes were predicted to be targeted from one or multiple DEmiRNAs from one or all queried reference databases. While the potential gene targets for miRNA can be numerous, we have ranked the importance of these interactions based on whether a specific interaction is found in more than one database.

**Figure 3.**
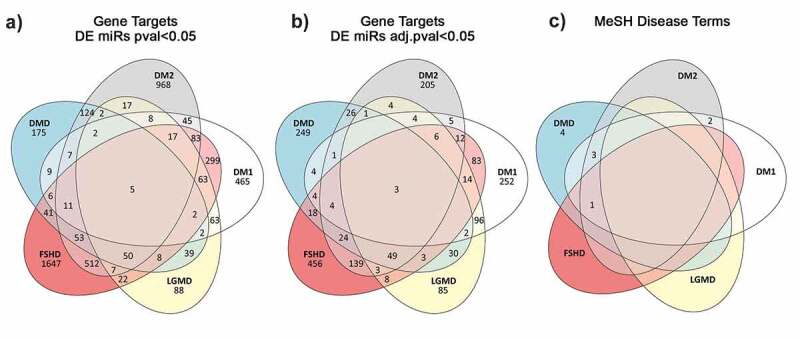
**Venn diagram showing the number of unique and common predicted miRNA target genes annotated with MeSH disease terms relevant to muscular dystrophies**. A) Gene targets predicted from the significantly DEmiRNAs (p-value <0.05) per disease. B) Gene targets predicted from the significantly DEmiRNAs after adjusting for multiple testing (adjusted p-value <0.05). C) Disease specific Medical Subject Headings associated with the gene targets predicted from the miR set identified after p-value adjustment.

The total predicted targets resulting from these analyses for DMD, DM1, DM2, FSHD1 and LGMD R1 calpain3-related amounted to 418, 490, 486, 826 and 308 genes, respectively. A summary of the common gene targets predicted across the five muscular dystrophies is shown in Fig. 3 A and B while the Supplementary Tables S13-16 show the top 20 genes per muscular dystrophy that are potential targets of the DEmiRNAs with an FDR value <0.05. The full datasets are given in the Supplementary Table S17.

### Functional analysis

The predicted gene targets were screened for known associations with diseases based on the Medical Subject Headings (MeSH) thesaurus. Only a relatively small number of genes were matched with MeSH terms related to muscular dystrophies. The number of unique and shared genes with a known association with muscular dystrophies is given in the Venn diagram shown in Fig. 3C.

**Figure 4.**
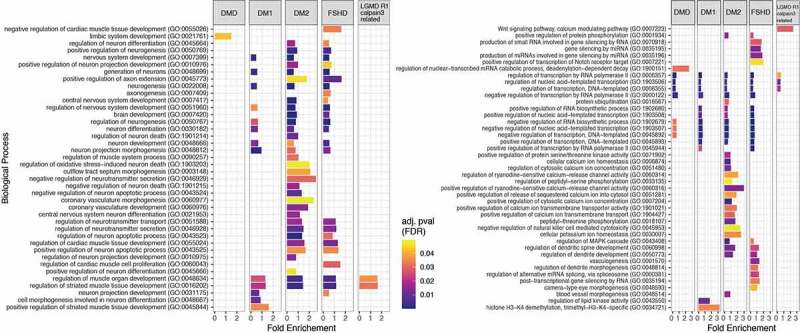
**Enriched biological processes based on the predicted target genes for each disease. Bar dimensions represent log (Fold Enrichment score) obtained for each process**. Bar colour represents the adjusted p-value with blue being of high significance and yellow close to the 0.05 adj. p-value threshold. The left panel are the processes related to muscle and neuronal activity while the right panel shows other identified processes.

To investigate further possible pathways through which the set of predicted target genes may be involved in muscular dystrophies we performed functional analysis using the PANTHER Classification System through the Gene Ontology web server [61,62] as described in Material and Methods. The functional analysis aimed at identifying the most relevant GO biological processes (BP) in which the predicted target genes are involved. Fig. 4 shows the most statistically significant biological processes in which the predicted genes are involved from GO BP levels 4 up to 16.

## Discussion

Muscular dystrophies are a group of inherited disorders that mainly affect the skeletal muscle tissues. Very frequently, the symptoms that the muscular dystrophy patients develop have an impact on the quality of life of the patients and on their life expectancy. Muscular dystrophies are a very heterogenic group of disorders in response to clinical phenotype, age of onset, affected organs and rate of progression. Circulating extracellular RNAs, including small RNA molecules, have been suggested as potential biomarkers for a wide range of diseases and other conditions [63]. In particular, miRNAs were found to stably circulate in the blood and were suggested as promising biomarkers for the diagnosis, prognosis or monitor of various diseases [40,64,65]. Up to date, targeted approaches have been mostly performed regarding the investigation of miRNAs as biomarkers for muscular dystrophies. Muscular dystrophies are rare disorders owing to limited work regarding the development of biomarkers especially using high-throughput methods. In this study, we provide the first genome-wide small RNA NGS analysis, to identify of the small RNA molecules circulating in the blood of patients affected by five different types of muscular dystrophies, DMD, DM1, DM2, FSHD1 and LGMD R1 calpain3-related.

Through the analysis of the small RNA content circulating in the serum of the patients, we showed that each type of muscular dystrophy has a distinct signature of circulating small RNA molecules. The DM2 patients who have the mildest symptoms compared to the patients affected by the other four muscular dystrophies, were found to have the highest percentage of lncRNAs and the lowest percentage of the snoRNAs and miRNAs compared to the other four muscular dystrophies. On the other hand, DMD patients presented the lowest percentage of lncRNAs and the highest percentage of miRNAs and snoRNAs compared to the other muscular dystrophy patients. These two types of small RNA molecules, snoRNAs and miRNAs, were previously reported to be related with muscle wasting [66]. Considering that DMD is a much more severe form of muscular dystrophy compared to DM2, the levels of these two types of small RNA molecules may reflect the severity of the symptoms of the patients. By comparing the two types of myotonic dystrophy, DM1 and DM2, we show that the DM1 patients have higher percentage of miRNAs compared to the DM2 although the percentage of circulating miRNAs is not as high as in DMD patients. LGMD R1 calpain3-related is also a very severe form of muscular dystrophy, usually slightly milder than DMD and affected individuals may remain ambulant into their twenties or even beyond. The patients affected by LGMD R1 calpain3-related, were found to have a higher percentage of miRNAs than the DM1 patients and a lower percentage of miRNAs than DMD patients. Furthermore, the percentage of miRNAs in FSHD1 patients was determined to be similar to that of DM1 patients. These two types of muscular dystrophies share very similar clinical characteristics and degree of severity [67]. These results suggest that the percentage of miRNAs circulating in the blood of muscular dystrophy patients is possibly correlated to the disease’s severity. Moreover, DM1 patients were identified to have a lower percentage of lncRNAs compared to DM2 patients who were found to have the highest percentage of lncRNAs amongst all the types of muscular dystrophies investigated. These observations imply that in myotonic dystrophies, the levels of miRNAs and lncRNAs circulating in the patients’ serum may reflect the severity of the symptoms between DM1 and DM2.

Following the screening of the entire small RNA population in all the five types of muscular dystrophies, we focused on the circulating miRNAs due to their high biological importance and the intense interest surrounding their use as biomarkers in many diseases including the muscular dystrophies [28,57]. Regarding muscular dystrophies, emphasis has been placed on the four myomiRs, miR-1, miR-133a, miR-133b and miR-206, for their potential use as biomarkers for some of the types of muscular dystrophies including DMD, DM1 and DM2 [43,68]. Notably, the myomiR levels were reported to be correlated with the disease severity and clinical assessments of the DMD patients and the progression of DM1 patients [42,56,68]. Other sporadic studies have reported additional miRNA candidate biomarkers for DMD, DM1 and DM2 [57,69]. Following small RNA NGS analysis, we identified a list of miRNAs with significantly differential serum levels in muscular dystrophy patients compared to controls and we next narrowed down the list of the top-scored miRNAs based on FDR analysis. The PCA plots showed that the top identified miRNAs can be used to distinguish the diseased group from the healthy participants. Various miRNAs were found to be altered in muscular dystrophy patients compared to healthy individuals (Figure S2). Some of the identified altered miRNAs are common across the patients of different types of muscular dystrophies while others are disease-specific. This finding enhances the possibility to find a good biomarker for the muscular dystrophies as a group of disorders. The miRNAs that were found to be increased in only one type of muscular dystrophy patients compared to healthy participants can be considered as candidate biomarkers to distinguish the patients of muscular dystrophies from healthy participants and also from the patients affected with other types of muscular dystrophies.

Furthermore, gene target prediction analysis was performed to provide the gene targets of the identified altered miRNAs for each of the five diseases investigated. A total of 418, 490, 486, 826 and 308 genes were predicted as targets for DMD, DM1, DM2, FSHD1 and LGMD R1 calpain3-related, respectively. Three of these genes, *ETS1, NR3C1* and *JARID2*, are common in all five muscular dystrophies with no previous association with any of the five muscular dystrophies based on bibliography. In addition, 249 disease-specific gene targets were found for DMD, 252 for DM1, 205 for DM2, 456 for FSHD1 and 85 for LGMD R1 calpain3-related. From the identified disease-specific genes, four genes (*ANK3, GALK2, ZBTB45* and *PLXNA1*) were previously reported to be involved in the pathogenesis of DMD and Becker Muscular Dystrophy (BMD), a milder form of DMD [70,71]. In agreement with the literature, we found that *PLXNA1* gene is a target of the altered miRNAs in DMD patients. The other three genes, *ANK3, GALK2* and *ZBTB45*, that we identified were previously reported to be associated with DMD however, our results show that these three genes are targets of the miRNAs that were found to be altered in DM1 patients. The gene targets that were predicted from the bioinformatics analysis of the altered miRNAs and are not disease-specific (166 for DMD, 235 for DM1, 278 for DM2, 367 for FSHD1 and 220 for LGMD R1 calpain3-related) were found in at least two of the five muscular dystrophies investigated. Of these, two genes, *CALU* and *PTORPG*, that were predicted as targets of the dysregulated miRNAs in both DM1 and DM2, three genes *PKHD1, TBC1D25* and *MRPS5*, that were predicted as targets of the dysregulated miRNAs in DM1, DM2 and DMD and one gene, *BCAT1*, that was predicted as target of the dysregulated miRNAs in the four types of muscular dystrophies analysed, DM1, DM2, DMD and FSHD1, were previously described to be involved to the corresponding muscular dystrophies [72–74].

Functional analysis was also performed to characterize the gene targets according to their biological role. Some of them were reported to be strongly related with muscle development such as the regulation of muscle organ development and the regulation of the striated muscle tissue development which were found to be common in the four out of five muscular dystrophies (DM1, DM2, FSHD1 and LGMD R1 calpain3-related), where the muscle is the main organ affected (Fig. 4). Although in DMD muscle tissue is also primarily affected, none of the gene targets identified is linked to pathways relating to muscle. Moreover, biological process related with the nervous system, such as nervous system development and generation of neurons were found to be common in only three muscular dystrophies, DM1, DM2 and FSHD1. Myotonic dystrophies, DM1 and DM2, are multisystemic diseases characterized not only by muscle dysfunction but also by central nervous system (CNS) alteration [75]. Symptoms can include cognitive and behavioural abnormalities and lack of executive function. The gene targets of the altered miRNAs were found to be involved in the function and development of the CNS in agreement with the multisystemic properties of myotonic dystrophies. miRNAs could therefore be involved in the formation of secondary symptoms such as nervous system related symptoms in the myotonic dystrophies, DM1 and DM2. Furthermore, CNS is also affected in FSHD1 patients who suffered with symptoms such as impairments in attention and memory [76]. This implies that miRNAs and their targets may be also involved in the development of secondary symptoms in FSHD1 patients. Remarkably in the identified biological processes, the limbic system development process was found to be involved in DMD patients only. Previous reports have described a poor activation of the limbic system in DMD patients compared to healthy individuals [77]. The limbic system is a set of structures in the brain that deal with emotions and memory. It regulates autonomic or endocrine function in response to emotional stimuli [78]. It has been previously reported that DMD patients develop behaviour and memory problems [79,80]. These symptoms are a consequence of the limbic system impairments thus explaining the altered levels of the specific miRNAs, and thereby their target genes, in the blood of DMD patients. Additional biological processes with no previous correlation to neuromuscular diseases were also identified, including the regulation of DNA transcription which was found in all the five muscular dystrophies. Our results suggest that this biological process could be involved in the pathogenesis of muscular dystrophies. Considering the high importance of this process in living cells, it would be of great interest to be further investigated.

In conclusion, our work examines for the first time the small RNA profile of muscular dystrophy patients and healthy individuals, for the discovery of small RNAs and pathways associated with the disease phenotype. Due to the rarity of muscular dystrophies, a limited sample size of eight patients was analysed for each type of muscular dystrophy. Although additional studies should be performed in a larger sample size, to further support the implication of the identified small RNAs and pathways to this group of diseases, this study reports important findings that could be used in the determination of the pathogenic mechanisms in each type of muscular dystrophy, as well as in the discovery of disease biomarkers. In particular, our results show that the patients affected with different types of muscular dystrophies are characterized by a unique signature of circulating small RNA molecules that can distinguish them from healthy individuals and that can help to classify patients into each type of muscular dystrophy. Furthermore, we provided an in-depth analysis of the miRNAs that are circulating in the blood of muscular dystrophy patients, through the characterization of their gene targets and associated pathways. These results provide significant evidence on the involvement of small RNAs in the pathogenesis of muscular dystrophies and the development of the symptoms and come from an easily accessible tissue, therefore providing valuable information for the development of miRNA disease biomarkers in future studies. Finally, our results provide a significant evidence of the involvement of the small RNA molecules in the pathogenesis of muscular dystrophies and the development of the symptoms.

## Materials and methods

### Participant inclusion, blood collection and isolation of serum

The study was approved by the National Bioethics Committees of the participating organizations and participants provided a written informed consent to participate and provide blood specimens to the study. The healthy participants did not have a family history of muscle disease. Following clinical examination, a total of 4 ml of blood was drawn from all study participants and placed in plain serum collection tubes (BD Vacutainer). Blood collection for miRNA analysis was performed following the last clinical examination of the patients. Serum was subsequently isolated from the blood samples.

### Small RNA isolation

Following serum collection, total RNA, including miRNAs, was extracted from serum samples using the miRNeasy Mini Kit (Qiagen), according to the manufacturer’s instructions.

### Small RNA-sequencing of miRNA population in serum of muscular dystrophy patients

Libraries were prepared from 5 μl of RNA using QIAseq miRNA Library Kit (Qiagen) following the manufacturers’ instructions. Library concentrations were measured using Qubit™ dsDNA HS Assay Kit (Thermo Fisher Scientific). Quality and concentration of libraries were determined by Real-Time PCR. Libraries were sequenced on a NextSeq 500 System (Illumina).

### Data preprocessing

Raw data were obtained from small RNA Next-Generation Sequencing (NGS). The raw data were processed for adaptor filtering and UMI-demultiplexing using the Trimommatic version 0.38 [81] and UMI-tools version 1.0.0 [82] in command line and were assessed for quality control using the FastQC version 0.11.5 [83].

### Mapping and quantification of reads

Mapping of raw reads was performed to the Genome Reference Consortium GRCh37 using Bowtie1 version 1.2.2 [84]. and mapping statistics were generated (Table S18). Quantification of mapped reads was performed using the HTSEQ-count tool [85]. All the mature miRNAs were quantified according to the General Feature Format (GFF) file that was retrieved from miRBase database in July 2019 [86].

### Differential expression analysis

Differential expression analysis was performed using the EdgeR package version 3.8 of R Bioconductor [59] for the identification of differentially expressed miRNAs (DEmiRNAs) between patient and control samples. The miRNA count matrices were normalized for RNA composition between libraries using a trimmed mean of M-values (TMM) normalization [87]. We kept the miRNAs with a minimum requirement of 1 count per million (CPM) across two or more libraries. The Quasi-Likelihood F-test (QLF) [88] was used as a statistical method to calculate the DEmiRNAs provided by the EdgeR package. Comparisons were made between patient and control samples for each of the five muscular dystrophies analysed: DM1, DM2, DMD, FSHD1 and LGMD R1 calpain3-related. The edgeR analysis returned the following: log_2_FC, logCPM, p-value, False Discovery Rate (FDR) and F-value for each miRNA (Supplementary tables S2-S6). The log_2_FC represents the Log_2_ Fold Change in the expression of each miRNA in patients compared to control samples, while the logCPM represents the log Counts Per Million as a measure of the miRNA’s expression level. The F-value was also calculated as the critical value of the QL F-test, based on which the p-value and FDR were estimated. A cut-off of p-value = 0.05 was used. Small RNA sequencing data is available through the public database European Nucleotide Archive (ENA) (https://www.ebi.ac.uk/ena/browser/home) supported by the European Bioinformatics Institute (EBI), under the following accession: PRJEB48580.

### Machine learning flow chart

**Figure 5.**
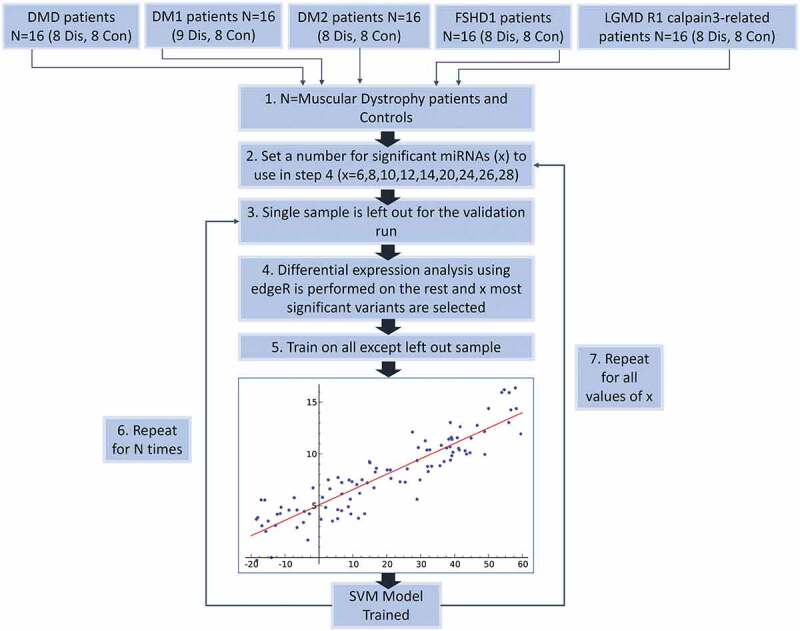
**Flow Chart of the classification process using a SVM classifier and edgeR as the feature (miRNA) selection methods**. Step 1 – Describes the dataset with two classes of patients from five different types of muscular dystrophy and their control samples. Step 2 – Denotes the selection of a set of miRNAs to be used during the leave-one-out classification (LOOCV) process. Step 3 – The LOOCV is initiated by extracting one sample from the dataset. Step 4 – edgeR is used to perform differential expression analysis on the remaining samples (this avoids overfitting). X number of top significant miRNAs are used for the next. Step 5 – A SVM model is trained using the data and features for the specific iteration. Step 6 – the LOOCV process (Steps 3–5) is repeated for every sample (N = #of muscular dystrophy patients) and statistics recorded. Step 7 – Steps 2–5 are repeated for every value of X.

The overall procedure of performing gene selection and further validation the selected genes using support vector machines (SVM) classification of disease control samples, is depicted in Fig. 5.

### Gene target prediction

The differentially expressed miRNA sets for each disease identified during the classification process were imported in Cytoscape v3.7.2 [89]. Using the ClueGO v2.5.5 [90]. and CluePedia v1.5.5 [91]. Each set was screened against a number of reference miRNA target edge lists that are available through CluePedia sourced from the databases shown in Table 2. Using CluePedia network extension function we constructed bipartite networks connecting each miRNA with their respective predicted gene targets using the default Kappa score for each edge list in Table 2 and an arbitrary limit of 50 genes per miRNA. Each network was stored in csv format for further processing in R. Since edges connecting a specific miRNA-gene pair may have been identified from more than one reference edge lists, using custom R scripts we recorded and ranked each pairwise connection (miRNA-Target gene) based on aggregated number of edges. For each disease the same process above was performed both for the entire set of miRNAs scoring a differential expression p-value <0.05 and for a conserved set of miRNAs that scored an FDR value of < 0.05. The latter set was selected for all downstream functional analysis.

**Table 2.** Source edge lists databases for miRNA – gene target predictions

Source DB	Database File	Edge List	Kappa Score Threshold
CluePedia	CluePedia_microRNA.org-human_predictions_S_C_aug2010.txt.gz	align_score/100_human_predictions_S_C_aug2010	0.6
miRDB [1]	miRDB_v6.0_prediction_hsa_based_on_miRBase_22_17.04.2019.txt.gz	miRanda-hsa-Score_miRDB_v6.0_prediction_based_on_miRBase_22	0.8
mirTarBase [2]	mirTarBase.validated.miRNAs_15.06.2016.txt	validated miRTarBase	0.6
miRecords [3]	mirecords.umn.edu.validated.miRNAs.2010–11-25.txt.gz	validated miRNA	0.6

**References**

1.Wong, N. and X. Wang, *miRDB: an online resource for microRNA target prediction and functional annotations*. Nucleic Acids Res, 2015. **43**(Database issue): p. D146-52.

2.Chou, C.H., *miRTarBase update 2018: a resource for experimentally validated microRNA-target interactions*. Nucleic Acids Res, 2018. **46**(D1): p. D296-D302.

3.Xiao, F., *miRecords: an integrated resource for microRNA-target interactions*. Nucleic Acids Res, 2009. **37**(Database issue): p. D105-10.

### Functional characterization

#### MeSH Disease term annotations

All predicted gene targets for the differentially expressed miRNAs were mapped to their respective Entrez IDs using Cytoscape’s *map column* function. Each gene Entrez ID was probed for disease terms in Medical Subject Headings (MeSH) [92] using the MetDisease v1.1.0 [93]. plugin in Cytoscape. Using custom R script, we queried the obtained tree structure terms to identify and record which of the predicted genes are associated with the five muscular dystrophies of interest.

#### GO Functional analysis

The predicted gene targets for each disease were used as an input in the GO enrichment analysis tool run by PANTHER Classification System server. Functional analysis was performed against the Homo Sapiens GO Biological Process database in order to obtain the terms that are related with the genes of interest. Statistical significance was determined using the Binomial Enrichment/Depletion (two-sided hypergeometric test) and a p-value threshold of <0.05 after Bonferroni step down correction.

## Supplementary Material

Supplemental Material
