# Supplementary material for: Circulating small RNA signatures differentiate accurately the subtypes of muscular dystrophies: small-RNA next-generation sequencing analytics and functional insights
Source: RNA Biol. 2022 Apr 7;19(1):507–18. doi: 10.1080/15476286.2022.2058817 (PMC8993092; doi:10.1080/15476286.2022.2058817)
Supplement: Supplemental Material [file KRNB_A_2058817_SM6377.zip › Supplementary Table S1.docx]

**Table S1: Summary of samples collected.**

| **Muscular Dystrophy** | **Patient** | **Gender** | **Age** |
| --- | --- | --- | --- |
| **DMD** | DMD 1 | MALE | 14 |
|  | DMD 2 | MALE | 15 |
|  | DMD 3 | MALE | 31 |
|  | DMD 4 | MALE | 20 |
|  | DMD 5 | MALE | 14 |
|  | DMD 6 | MALE | 15 |
|  | DMD 7 | MALE | 11 |
|  | DMD 8 | MALE | 6 |
| **DM1** | DM1 1 | MALE | 34 |
|  | DM1 2 | MALE | 37 |
|  | DM1 3 | FEMALE | 57 |
|  | DM1 4 | FEMALE | 57 |
|  | DM1 5 | FEMALE | 48 |
|  | DM1 6 | FEMALE | 42 |
|  | DM1 7 | FEMALE | 64 |
|  | DM1 8 | FEMALE | 30 |
| **DM2** | DM2 1 | MALE | 71 |
|  | DM2 2 | MALE | 69 |
|  | DM2 3 | FEMALE | 72 |
|  | DM2 4 | FEMALE | 59 |
|  | DM2 5 | FEMALE | 74 |
|  | DM2 6 | MALE | 50 |
|  | DM2 7 | MALE | 37 |
|  | DM2 8 | FEMALE | 60 |
| **FSHD1** | FSHD 1 | MALE | 60 |
|  | FHSD 2 | MALE | 70 |
|  | FHSD 3 | MALE | 32 |
|  | FSHD 4 | MALE | 57 |
|  | FSHD 5 | MALE | 57 |
|  | FSHD 6 | MALE | 50 |
|  | FSHD 7 | FEMALE | 65 |
|  | FSHD 8 | FEMALE | 36 |
| **LGMD R1 calpain3-related** | LGMD 1 | FEMALE | 53 |
|  | LGMD 2 | MALE | 50 |
|  | LGMD 3 | MALE | 26 |
|  | LGMD 4 | FEMALE | 65 |
|  | LGMD 5 | FEMALE | 33 |
|  | LGMD 6 | MALE | 31 |
|  | LGMD 7 | FEMALE | 41 |
|  | LGMD 8 | MALE | 34 |
| **Healthy** | Control 1 | MALE | 33 |
|  | Control 2 | MALE | 23 |
|  | Control 3 | MALE | 10 |
|  | Control 4 | MALE | 56 |
|  | Control 5 | MALE | 57 |
|  | Control 6 | FEMALE | 50 |
|  | Control 7 | FEMALE | 65 |
|  | Control 8 | MALE | 68 |
|  | Control 9 | MALE | 58 |
|  | Control 10 | MALE | 63 |
|  | Control 11 | MALE | 15 |
|  | Control 12 | MALE | 17 |
|  | Control 13 | FEMALE | 32 |
|  | Control 14 | MALE | 73 |
|  | Control 15 | FEMALE | 72 |
|  | Control 16 | FEMALE | 76 |
|  | Control 17 | MALE | 16 |
|  | Control 18 | MALE | 12 |
|  | Control 19 | FEMALE | 59 |
|  | Control 20 | FEMALE | 39 |
|  | Control 21 | MALE | 6 |
|  | Control 22 | FEMALE | 46 |
