# Supplementary material for: Circulating small RNA signatures differentiate accurately the subtypes of muscular dystrophies: small-RNA next-generation sequencing analytics and functional insights
Source: RNA Biol. 2022 Apr 7;19(1):507–18. doi: 10.1080/15476286.2022.2058817 (PMC8993092; doi:10.1080/15476286.2022.2058817)
Supplement: Supplemental Material [file KRNB_A_2058817_SM6377.zip › Supplementary Table S10.docx]

**Table S10. DM2 LOOCV panel of pooled top scoring miRNAs.**

|  | **logFC** | **logCPM** | **F** | **P-Value** | **FDR** |
| --- | --- | --- | --- | --- | --- |
| hsa-miR-106b-3p | -1.63733 | 8.50629 | 11.299 | 0.000975 | 0.096688 |
| hsa-miR-1268b | 3.433592 | 2.243158 | 8.750302 | 0.003101 | 0.148417 |
| hsa-miR-1291 | 4.25993 | 2.390225 | 9.693032 | 0.001861 | 0.125288 |
| hsa-miR-133a | -2.68756 | 5.170799 | 9.711267 | 0.001835 | 0.112608 |
| hsa-miR-142-5p | -1.34167 | 10.24441 | 10.09623 | 0.001667 | 0.107547 |
| hsa-miR-1468 | -4.80886 | 3.040621 | 9.473482 | 0.002101 | 0.121724 |
| hsa-miR-16-5p | -0.97699 | 18.61253 | 10.28023 | 0.001348 | 0.088748 |
| hsa-miR-199b-5p | 3.560435 | 4.670941 | 12.10266 | 0.000505 | 0.128557 |
| hsa-miR-203 | 1.731663 | 7.587139 | 10.12139 | 0.00151 | 0.104718 |
| hsa-miR-206 | 2.522539 | 8.817449 | 23.7495 | 1.11E-06 | 0.0011 |
| hsa-miR-210 | -5.09364 | 4.098297 | 14.65087 | 0.000164 | 0.065418 |
| hsa-miR-214-3p | 3.925931 | 3.496562 | 9.806278 | 0.001835 | 0.116466 |
| hsa-miR-22-5p | -5.28934 | 3.136782 | 10.59801 | 0.001211 | 0.114056 |
| hsa-miR-221-3p | -1.42342 | 8.957151 | 9.627963 | 0.001949 | 0.121897 |
| hsa-miR-221-5p | 4.10988 | 3.306086 | 9.299922 | 0.002296 | 0.137399 |
| hsa-miR-25-3p | -1.53947 | 12.42192 | 15.24939 | 0.000188 | 0.066353 |
| hsa-miR-296-5p | -4.91308 | 2.942966 | 9.172634 | 0.002463 | 0.119822 |
| hsa-miR-29c-5p | -5.34923 | 3.232315 | 10.20633 | 0.001403 | 0.112608 |
| hsa-miR-30e-5p | -1.63547 | 9.842865 | 13.55112 | 0.000363 | 0.076545 |
| hsa-miR-3120-5p | 3.973584 | 3.488931 | 9.92567 | 0.001729 | 0.117431 |
| hsa-miR-3200-3p | -5.51383 | 3.295746 | 11.87712 | 0.000634 | 0.100023 |
| hsa-miR-320c | 1.502745 | 9.347123 | 10.84359 | 0.000994 | 0.110998 |
| hsa-miR-3545-5p | 1.731663 | 7.587139 | 10.12139 | 0.00151 | 0.104718 |
| hsa-miR-362-3p | 4.169011 | 2.430188 | 9.54037 | 0.002017 | 0.119718 |
| hsa-miR-363-3p | -1.94596 | 7.414982 | 12.36415 | 0.000568 | 0.089643 |
| hsa-miR-3688-3p | -4.98807 | 3.037047 | 9.889896 | 0.001741 | 0.117932 |
| hsa-miR-3688-5p | -4.98807 | 3.037047 | 9.889896 | 0.001741 | 0.117932 |
| hsa-miR-369-5p | 4.44602 | 3.423198 | 10.6144 | 0.001125 | 0.128557 |
| hsa-miR-425-5p | -1.34708 | 10.6135 | 10.53411 | 0.001319 | 0.107547 |
| hsa-miR-4426 | 4.297714 | 2.463326 | 9.668845 | 0.001882 | 0.115568 |
| hsa-miR-451a | -1.22351 | 13.77684 | 10.88938 | 0.00097 | 0.079202 |
| hsa-miR-4685-5p | 3.532996 | 2.256226 | 8.973667 | 0.002806 | 0.130513 |
| hsa-miR-4732-3p | -1.91078 | 6.788861 | 10.36928 | 0.001455 | 0.106649 |
| hsa-miR-4755-5p | -4.05385 | 3.251194 | 8.854737 | 0.002929 | 0.137399 |
| hsa-miR-487b | -4.79052 | 2.923716 | 8.849535 | 0.002939 | 0.119822 |
| hsa-miR-5003-3p | 4.465838 | 2.619654 | 9.768244 | 0.001877 | 0.116934 |
| hsa-miR-505-5p | -5.51153 | 4.672984 | 19.51246 | 1.01E-05 | 0.009945 |
| hsa-miR-548i | 3.959933 | 2.34908 | 9.355729 | 0.002233 | 0.125326 |
| hsa-miR-576-5p | -2.19935 | 6.044083 | 10.43447 | 0.00145 | 0.104973 |
| hsa-miR-642a-3p | -5.0726 | 3.044621 | 9.877526 | 0.001765 | 0.12127 |
| hsa-miR-642b-5p | -5.0726 | 3.044621 | 9.877526 | 0.001765 | 0.12127 |
| hsa-miR-769-5p | -5.32021 | 3.131097 | 10.39822 | 0.001264 | 0.123681 |
| hsa-miR-942 | -3.0816 | 5.27597 | 12.44952 | 0.00042 | 0.050146 |
| hsa-miR-96-5p | -2.11917 | 6.23805 | 10.49623 | 0.001379 | 0.101074 |
