# Supplementary material for: Circulating small RNA signatures differentiate accurately the subtypes of muscular dystrophies: small-RNA next-generation sequencing analytics and functional insights
Source: RNA Biol. 2022 Apr 7;19(1):507–18. doi: 10.1080/15476286.2022.2058817 (PMC8993092; doi:10.1080/15476286.2022.2058817)
Supplement: Supplemental Material [file KRNB_A_2058817_SM6377.zip › Supplementary Table S11.docx]

**Table S11. FSHD1 LOOCV panel of pooled top scoring miRNAs.**

|  | **logFC** | **logCPM** | **F** | **P-Value** | **FDR** |
| --- | --- | --- | --- | --- | --- |
| hsa-let-7a-3p | 4.860423 | 2.813016 | 10.07056 | 0.001635 | 0.115644 |
| hsa-let-7a-5p | -1.01253 | 15.64275 | 9.850163 | 0.001702 | 0.101917 |
| hsa-miR-100-5p | 1.731799 | 6.479467 | 9.225952 | 0.002391 | 0.153556 |
| hsa-miR-1238 | -3.34034 | 2.202183 | 8.67166 | 0.003338 | 0.125866 |
| hsa-miR-125a-3p | -3.97714 | 2.558744 | 8.644935 | 0.003286 | 0.140103 |
| hsa-miR-127-3p | 2.333976 | 5.333399 | 11.17737 | 0.001115 | 0.097602 |
| hsa-miR-1277-5p | -3.72643 | 3.757094 | 11.2973 | 0.001004 | 0.096602 |
| hsa-miR-1296 | 4.022912 | 2.48927 | 8.147168 | 0.004339 | 0.14761 |
| hsa-miR-1299 | 3.931087 | 3.228748 | 8.324467 | 0.00399 | 0.146006 |
| hsa-miR-144-5p | -1.53389 | 7.595315 | 8.840391 | 0.002952 | 0.146795 |
| hsa-miR-150-5p | 1.247944 | 11.18507 | 8.940236 | 0.002795 | 0.10496 |
| hsa-miR-16-5p | -0.91002 | 18.5355 | 9.337642 | 0.002254 | 0.133266 |
| hsa-miR-17-3p | 4.195429 | 2.532176 | 8.408788 | 0.003823 | 0.145664 |
| hsa-miR-186-3p | -3.34605 | 2.24525 | 8.478284 | 0.003793 | 0.138645 |
| hsa-miR-188-5p | -3.48426 | 2.271514 | 8.090404 | 0.004458 | 0.13616 |
| hsa-miR-196a-5p | -1.99568 | 5.571142 | 9.077063 | 0.002982 | 0.124508 |
| hsa-miR-197-5p | -4.15588 | 2.612857 | 8.810215 | 0.003001 | 0.146795 |
| hsa-miR-199b-5p | 3.595472 | 4.439363 | 15.52854 | 0.00017 | 0.036433 |
| hsa-miR-206 | 2.429598 | 8.763306 | 25.08658 | 1.29E-06 | 0.000942 |
| hsa-miR-218-2-3p | -3.44621 | 2.250062 | 8.060603 | 0.004532 | 0.13616 |
| hsa-miR-221-5p | 4.05065 | 3.522707 | 10.40662 | 0.001259 | 0.099678 |
| hsa-miR-223-3p | 1.722026 | 12.94713 | 19.45377 | 3.35E-05 | 0.014826 |
| hsa-miR-2355-3p | -3.95451 | 2.429417 | 9.173311 | 0.002658 | 0.130155 |
| hsa-miR-27a-5p | 5.46998 | 3.239031 | 12.38468 | 0.000435 | 0.05109 |
| hsa-miR-28-5p | 4.930366 | 2.898329 | 9.907866 | 0.00165 | 0.104522 |
| hsa-miR-296-3p | -3.67401 | 2.429857 | 8.097099 | 0.004441 | 0.169476 |
| hsa-miR-3115 | -3.47823 | 2.315607 | 7.796849 | 0.005242 | 0.137039 |
| hsa-miR-3127-3p | -3.43349 | 2.266845 | 8.529778 | 0.003684 | 0.140276 |
| hsa-miR-3161 | -3.54277 | 2.434671 | 7.886821 | 0.004988 | 0.146795 |
| hsa-miR-3188 | -3.29251 | 2.236029 | 7.65994 | 0.005655 | 0.137039 |
| hsa-miR-3193 | -4.71655 | 2.75225 | 11.44178 | 0.00082 | 0.098477 |
| hsa-miR-337-5p | -3.91057 | 2.405536 | 9.098662 | 0.002774 | 0.129646 |
| hsa-miR-3605-5p | -3.6448 | 3.403459 | 8.395774 | 0.003768 | 0.146795 |
| hsa-miR-362-3p | 3.786587 | 2.445957 | 7.813986 | 0.005279 | 0.148873 |
| hsa-miR-369-5p | 4.797704 | 2.832853 | 9.325982 | 0.002264 | 0.10553 |
| hsa-miR-370 | 3.24403 | 4.118125 | 10.20347 | 0.001405 | 0.091153 |
| hsa-miR-382-3p | 4.046825 | 2.503878 | 8.173386 | 0.004297 | 0.145088 |
| hsa-miR-409-3p | 1.761375 | 6.633217 | 10.02517 | 0.001811 | 0.103649 |
| hsa-miR-431-5p | 2.386427 | 5.173505 | 11.36696 | 0.002048 | 0.07957 |
| hsa-miR-432-5p | 1.479584 | 7.656193 | 8.7444 | 0.003112 | 0.111774 |
| hsa-miR-4429 | -4.28184 | 2.855602 | 9.391923 | 0.002184 | 0.132126 |
| hsa-miR-4435 | -4.16753 | 2.421562 | 9.570723 | 0.001982 | 0.129077 |
| hsa-miR-4446-3p | 5.593069 | 3.383233 | 13.59655 | 0.000228 | 0.034446 |
| hsa-miR-449a | -3.83079 | 2.356145 | 8.969888 | 0.002993 | 0.128201 |
| hsa-miR-4511 | -3.89491 | 2.508332 | 8.414148 | 0.00373 | 0.146795 |
| hsa-miR-4518 | -3.2703 | 2.187029 | 8.59764 | 0.003428 | 0.132029 |
| hsa-miR-4662a-5p | -3.17967 | 2.179704 | 8.537327 | 0.003512 | 0.137898 |
| hsa-miR-4662b | -3.18917 | 2.192555 | 8.599952 | 0.003383 | 0.139368 |
| hsa-miR-4677-3p | -3.08231 | 2.162078 | 8.414802 | 0.003754 | 0.143116 |
| hsa-miR-4683 | -4.64565 | 2.710389 | 11.07892 | 0.000978 | 0.103627 |
| hsa-miR-4755-3p | -4.95835 | 2.801747 | 12.30864 | 0.000519 | 0.076485 |
| hsa-miR-4755-5p | -4.2297 | 2.82599 | 9.086309 | 0.002892 | 0.119172 |
| hsa-miR-493-3p | 4.092116 | 2.517725 | 8.180685 | 0.004272 | 0.144525 |
| hsa-miR-4999-5p | -3.06752 | 2.17697 | 8.440308 | 0.003688 | 0.140456 |
| hsa-miR-499a-5p | 5.158373 | 2.904711 | 11.12911 | 0.001024 | 0.09431 |
| hsa-miR-499b-3p | 5.158373 | 2.904711 | 11.12911 | 0.001024 | 0.09431 |
| hsa-miR-500b | 3.671742 | 2.4747 | 7.53303 | 0.006067 | 0.161001 |
| hsa-miR-524-3p | -3.51757 | 2.227408 | 8.374949 | 0.003811 | 0.132126 |
| hsa-miR-5586-3p | -2.99131 | 2.153794 | 8.203379 | 0.00419 | 0.154855 |
| hsa-miR-5590-3p | -3.08853 | 2.154615 | 8.471198 | 0.003633 | 0.139619 |
| hsa-miR-5697 | -3.97955 | 2.416928 | 9.23583 | 0.002603 | 0.130313 |
| hsa-miR-618 | 3.679093 | 2.469816 | 7.8294 | 0.005149 | 0.140103 |
| hsa-miR-624-5p | -2.93373 | 2.147538 | 8.172992 | 0.004288 | 0.15139 |
| hsa-miR-627 | -3.583 | 2.274569 | 8.692047 | 0.003426 | 0.135244 |
| hsa-miR-676-3p | 3.732559 | 2.389615 | 7.685966 | 0.005574 | 0.169476 |
| hsa-miR-7-1-3p | 3.836523 | 2.491937 | 7.871176 | 0.005069 | 0.146048 |
| hsa-miR-769-5p | 4.106641 | 3.946974 | 14.37659 | 0.00015 | 0.027013 |
| hsa-miR-877-5p | 4.27094 | 2.594309 | 8.601396 | 0.003449 | 0.145029 |
| hsa-miR-9-3p | -4.29645 | 2.983194 | 9.826738 | 0.002215 | 0.111562 |
