# Supplementary material for: Circulating small RNA signatures differentiate accurately the subtypes of muscular dystrophies: small-RNA next-generation sequencing analytics and functional insights
Source: RNA Biol. 2022 Apr 7;19(1):507–18. doi: 10.1080/15476286.2022.2058817 (PMC8993092; doi:10.1080/15476286.2022.2058817)
Supplement: Supplemental Material [file KRNB_A_2058817_SM6377.zip › Supplementary Table S12.docx]

**Table S12. LGMD R1 calpain3-related LOOCV panel of pooled top scoring miRNAs.**

|  | **logFC** | **logCPM** | **F** | **P-Value** | **FDR** |
| --- | --- | --- | --- | --- | --- |
| hsa-miR-142-3p | 2.587385 | 12.30987 | 30.58058 | 5.55E-07 | 0.000164 |
| hsa-miR-143-3p | 2.582638 | 10.71116 | 27.19168 | 1.33E-06 | 0.000301 |
| hsa-miR-206 | 3.996412 | 9.562279 | 43.11261 | 5.39E-11 | 4.77E-08 |
| hsa-miR-208b | 6.585031 | 4.170267 | 21.26204 | 7.55E-06 | 0.00116 |
| hsa-miR-223-3p | 2.196852 | 14.11149 | 26.00533 | 1.49E-06 | 0.000363 |
| hsa-miR-4418 | -6.43861 | 3.830029 | 19.64393 | 1.08E-05 | 0.001911 |
| hsa-miR-486-3p | -1.876 | 16.38273 | 22.28071 | 6.02E-06 | 0.001021 |
| hsa-miR-486-5p | -1.93857 | 16.64457 | 23.76916 | 3.10E-06 | 0.000674 |
