# Supplementary material for: Circulating small RNA signatures differentiate accurately the subtypes of muscular dystrophies: small-RNA next-generation sequencing analytics and functional insights
Source: RNA Biol. 2022 Apr 7;19(1):507–18. doi: 10.1080/15476286.2022.2058817 (PMC8993092; doi:10.1080/15476286.2022.2058817)
Supplement: Supplemental Material [file KRNB_A_2058817_SM6377.zip › Supplementary Table S13.docx]

**Table S13. Top 20 predicted gene targets by differentially expressed miRNAs in DMD.**

|  | **DMD** | | | | | | | |
| --- | --- | --- | --- | --- | --- | --- | --- | --- |
| **Gene ID** | **hsa-miR** | | | | | | **Total Edges** | **Total miRNAs** |
|  | **1** | **133a** | **203** | **206** | **3131** | **3545-5p** |  |  |
| **ZNF800** | 4 | 2 | 3 | 4 | 0 | 1 | 14 | 5 |
| **PHF6** | 4 | 0 | 4 | 4 | 0 | 1 | 13 | 4 |
| **MFSD14A** | 4 | 0 | 3 | 4 | 0 | 1 | 12 | 4 |
| **UTRN** | 5 | 0 | 2 | 5 | 0 | 0 | 12 | 3 |
| **ADAM12** | 4 | 1 | 3 | 3 | 0 | 0 | 11 | 4 |
| **BCL7A** | 3 | 0 | 4 | 3 | 1 | 0 | 11 | 4 |
| **CAP1** | 5 | 3 | 0 | 3 | 0 | 0 | 11 | 3 |
| **ETS1** | 5 | 0 | 2 | 4 | 0 | 0 | 11 | 3 |
| **JARID2** | 4 | 0 | 3 | 4 | 0 | 0 | 11 | 3 |
| **MAP4K3** | 4 | 0 | 3 | 4 | 0 | 0 | 11 | 3 |
| **MED14** | 3 | 2 | 3 | 3 | 0 | 0 | 11 | 4 |
| **NETO2** | 5 | 0 | 3 | 3 | 0 | 0 | 11 | 3 |
| **PREX1** | 4 | 2 | 3 | 2 | 0 | 0 | 11 | 4 |
| **PTPRG** | 4 | 0 | 3 | 4 | 0 | 0 | 11 | 3 |
| **CDK14** | 4 | 0 | 2 | 4 | 0 | 0 | 10 | 3 |
| **CLCN3** | 6 | 0 | 0 | 4 | 0 | 0 | 10 | 2 |
| **CPEB1** | 4 | 0 | 2 | 4 | 0 | 0 | 10 | 3 |
| **DDX5** | 6 | 0 | 0 | 4 | 0 | 0 | 10 | 2 |
| **EPHB1** | 4 | 0 | 2 | 4 | 0 | 0 | 10 | 3 |
| **SLC35G1** | 4 | 0 | 2 | 4 | 0 | 0 | 10 | 3 |

The supplementary tables show the individual miRNA targeting each gene while the number in the cells corresponds to number of edges connecting each pair. The total number of edges of a specific gene (degree) and the number of different miRNAs targeting it are given in the last two columns, respectively.
