# Supplementary material for: Circulating small RNA signatures differentiate accurately the subtypes of muscular dystrophies: small-RNA next-generation sequencing analytics and functional insights
Source: RNA Biol. 2022 Apr 7;19(1):507–18. doi: 10.1080/15476286.2022.2058817 (PMC8993092; doi:10.1080/15476286.2022.2058817)
Supplement: Supplemental Material [file KRNB_A_2058817_SM6377.zip › Supplementary Table S14.docx]

**Table S14. Top 20 predicted gene targets by differentially expressed miRNAs in DM1 and DM2.**

| **DM1** | | | | | | | |  | **DM2** | |
| --- | --- | --- | --- | --- | --- | --- | --- | --- | --- | --- |
| **Gene ID** | **hsa-miR** | | | | | **Total Edges** | **Total miRNAs** |  | **Gene ID** | **hsa-miR-206** |
|  | **1277-5p** | **142-3p** | **4418** | **4446-3p** | **5187-5p** |  |  |  |  |  |
| **CPEB2** | 2 | 5 | 0 | 0 | 0 | 7 | 2 |  | **FRS2** | 5 |
| **C5orf24** | 0 | 5 | 1 | 0 | 0 | 6 | 2 |  | **RNF138** | 5 |
| **FMNL2** | 2 | 4 | 0 | 0 | 0 | 6 | 2 |  | **API5** | 4 |
| **GTF2A1** | 2 | 4 | 0 | 0 | 0 | 6 | 2 |  | **BHLHE22** | 4 |
| **ZCCHC14** | 0 | 5 | 0 | 1 | 0 | 6 | 2 |  | **CPEB1** | 4 |
| **ATF7IP** | 1 | 4 | 0 | 0 | 0 | 5 | 2 |  | **CREBL2** | 4 |
| **PHIP** | 2 | 3 | 0 | 0 | 0 | 5 | 2 |  | **DDX5** | 4 |
| **PIK3CG** | 1 | 4 | 0 | 0 | 0 | 5 | 2 |  | **EIF4E** | 4 |
| **RNF38** | 0 | 4 | 1 | 0 | 0 | 5 | 2 |  | **ETS1** | 4 |
| **SLC1A3** | 0 | 4 | 0 | 0 | 1 | 5 | 2 |  | **FAM91A1** | 4 |
| **TAB2** | 0 | 5 | 0 | 0 | 0 | 5 | 1 |  | **FBXL14** | 4 |
| **TET2** | 2 | 3 | 0 | 0 | 0 | 5 | 2 |  | **FBXW7** | 4 |
| **ZNF217** | 0 | 4 | 0 | 1 | 0 | 5 | 2 |  | **FNDC3B** | 4 |
| **ATP2A2** | 0 | 4 | 0 | 0 | 0 | 4 | 1 |  | **GLCCI1** | 4 |
| **CUL5** | 0 | 4 | 0 | 0 | 0 | 4 | 1 |  | **HSPD1** | 4 |
| **DIDO1** | 0 | 3 | 0 | 0 | 1 | 4 | 2 |  | **JARID2** | 4 |
| **DMRT2** | 1 | 3 | 0 | 0 | 0 | 4 | 2 |  | **LRRC59** | 4 |
| **FAM177A1** | 1 | 3 | 0 | 0 | 0 | 4 | 2 |  | **MAP4K3** | 4 |
| **FIGN** | 0 | 3 | 1 | 0 | 0 | 4 | 2 |  | **MFSD14A** | 4 |
| **HAPLN1** | 1 | 2 | 0 | 0 | 1 | 4 | 3 |  | **NAB1** | 4 |

The supplementary tables show the individual miRNA targeting each gene while the number in the cells corresponds to number of edges connecting each pair. The total number of edges of a specific gene (degree) and the number of different miRNAs targeting it are given in the last two columns, respectively.
