# Supplementary material for: Circulating small RNA signatures differentiate accurately the subtypes of muscular dystrophies: small-RNA next-generation sequencing analytics and functional insights
Source: RNA Biol. 2022 Apr 7;19(1):507–18. doi: 10.1080/15476286.2022.2058817 (PMC8993092; doi:10.1080/15476286.2022.2058817)
Supplement: Supplemental Material [file KRNB_A_2058817_SM6377.zip › Supplementary Table S15.docx]

**Table S15. Top 20 predicted gene targets by differentially expressed miRNAs in FSHD1.**

| **Gene ID** | **FSHD1** | | | | |
| --- | --- | --- | --- | --- | --- |
|  | **hsa-miR** | | |  |  |
|  | **206** | **4446-3p** | **769-5p** | **Total Edges** | **Total miRNAs** |
| **CPEB1** | 4 | 0 | 1 | 5 | 2 |
| **EIF1AX** | 5 | 0 | 0 | 5 | 1 |
| **EIF4E** | 4 | 0 | 1 | 5 | 2 |
| **FRS2** | 5 | 0 | 0 | 5 | 1 |
| **MAL2** | 4 | 1 | 0 | 5 | 2 |
| **PDCD4** | 4 | 0 | 1 | 5 | 2 |
| **RNF138** | 5 | 0 | 0 | 5 | 1 |
| **ANP32E** | 4 | 0 | 0 | 4 | 1 |
| **API5** | 4 | 0 | 0 | 4 | 1 |
| **BCL11A** | 4 | 0 | 0 | 4 | 1 |
| **BHLHE22** | 4 | 0 | 0 | 4 | 1 |
| **BPNT1** | 3 | 0 | 1 | 4 | 2 |
| **CHSY1** | 4 | 0 | 0 | 4 | 1 |
| **CLTC** | 4 | 0 | 0 | 4 | 1 |
| **DDX5** | 4 | 0 | 0 | 4 | 1 |
| **E2F5** | 4 | 0 | 0 | 4 | 1 |
| **ETS1** | 4 | 0 | 0 | 4 | 1 |
| **FAM91A1** | 4 | 0 | 0 | 4 | 1 |
| **FBXW7** | 4 | 0 | 0 | 4 | 1 |
| **FN1** | 4 | 0 | 0 | 4 | 1 |

The supplementary tables show the individual miRNA targeting each gene while the number in the cells corresponds to number of edges connecting each pair. The total number of edges of a specific gene (degree) and the number of different miRNAs targeting it are given in the last two columns, respectively.
