# Supplementary material for: Circulating small RNA signatures differentiate accurately the subtypes of muscular dystrophies: small-RNA next-generation sequencing analytics and functional insights
Source: RNA Biol. 2022 Apr 7;19(1):507–18. doi: 10.1080/15476286.2022.2058817 (PMC8993092; doi:10.1080/15476286.2022.2058817)
Supplement: Supplemental Material [file KRNB_A_2058817_SM6377.zip › Supplementary Table S16.docx]

**Table S16. Top 20 predicted gene targets by differentially expressed miRNAs in LGMD R1 calpain3-related.**

|  | **LGMD R1 calpain3-related** | | | | | | |
| --- | --- | --- | --- | --- | --- | --- | --- |
| **GENE ID** | **hsa-miR** | | | | | **Total Edges** | **Total miRNAs** |
|  | **142-3p** | **206** | **208b** | **4418** | **486-3p** |  |  |
| **BCL11A** | 2 | 4 | 0 | 1 | 1 | 8 | 4 |
| **ETS1** | 0 | 4 | 4 | 0 | 0 | 8 | 2 |
| **CUL5** | 4 | 0 | 3 | 0 | 0 | 7 | 2 |
| **DDX5** | 0 | 4 | 3 | 0 | 0 | 7 | 2 |
| **HMBOX1** | 2 | 3 | 2 | 0 | 0 | 7 | 3 |
| **JARID2** | 0 | 4 | 2 | 1 | 0 | 7 | 3 |
| **PIK3CG** | 4 | 3 | 0 | 0 | 0 | 7 | 2 |
| **PMS1** | 3 | 2 | 2 | 0 | 0 | 7 | 3 |
| **PSIP1** | 3 | 3 | 0 | 1 | 0 | 7 | 3 |
| **BCL2** | 2 | 3 | 0 | 0 | 1 | 6 | 3 |
| **C5orf24** | 5 | 0 | 0 | 1 | 0 | 6 | 2 |
| **E2F7** | 2 | 0 | 3 | 1 | 0 | 6 | 3 |
| **KIF20B** | 3 | 1 | 2 | 0 | 0 | 6 | 3 |
| **NLK** | 2 | 0 | 4 | 0 | 0 | 6 | 2 |
| **ANKFY1** | 0 | 3 | 2 | 0 | 0 | 5 | 2 |
| **ATP2A2** | 4 | 0 | 1 | 0 | 0 | 5 | 2 |
| **COG3** | 3 | 2 | 0 | 0 | 0 | 5 | 2 |
| **CRIM1** | 3 | 0 | 2 | 0 | 0 | 5 | 2 |
| **DMTF1** | 3 | 0 | 2 | 0 | 0 | 5 | 2 |
| **ERCC6L2** | 2 | 0 | 3 | 0 | 0 | 5 | 2 |

The supplementary tables show the individual miRNA targeting each gene while the number in the cells corresponds to number of edges connecting each pair. The total number of edges of a specific gene (degree) and the number of different miRNAs targeting it are given in the last two columns, respectively.
