# Supplementary material for: Circulating small RNA signatures differentiate accurately the subtypes of muscular dystrophies: small-RNA next-generation sequencing analytics and functional insights
Source: RNA Biol. 2022 Apr 7;19(1):507–18. doi: 10.1080/15476286.2022.2058817 (PMC8993092; doi:10.1080/15476286.2022.2058817)
Supplement: Supplemental Material [file KRNB_A_2058817_SM6377.zip › Supplementary Table S18.docx]

| **1^st^ run** | Sample-1 | Sample-2 | Sample-3 | Sample-4 | Sample-5 | Sample-6 | Sample-7 | Sample-8 |  |  |  |  |  |  |  |
| --- | --- | --- | --- | --- | --- | --- | --- | --- | --- | --- | --- | --- | --- | --- | --- |
| **Percentage mapping** | 100 | 100 | 100 | 100 | 100 | 100 | 100 | 100 |  |  |  |  |  |  |  |
| **Number of mapped reads** | 14802168 | 7695580 | 9587317 | 8702915 | 8196028 | 10206820 | 11892068 | 7529587 |  |  |  |  |  |  |  |
| **2^nd^ run** | Sample-B1 | Sample-B2 | Sample-B3 | Sample-B4 | Sample-B5 | Sample-B6 | Sample-B7 | Sample-B8 | Sample-B9 | Sample-B10 |  |  |  |  |  |
| **Percentage mapping** | 100 | 100 | 100 | 100 | 100 | 100 | 100 | 100 | 100 | 100 |  |  |  |  |  |
| **Number of mapped reads** | 12209569 | 11556513 | 10282284 | 9481357 | 10979459 | 7384017 | 8156651 | 7675684 | 7587195 | 7482523 |  |  |  |  |  |
| **3^rd^ run** | Sample-C1 | Sample-C2 | Sample-C3 | Sample-C4 | Sample-C5 | Sample-C6 | Sample-C7 | Sample-C8 | Sample-C9 | Sample-C10 | Sample-C11 | Sample-C12 |  |  |  |
| **Percentage mapping** | 100 | 100 | 100 | 100 | 100 | 100 | 100 | 100 | 100 | 100 | 100 | 100 |  |  |  |
| **Number of mapped reads** | 6071569 | 8183178 | 5458264 | 6225942 | 8460550 | 11164423 | 9455183 | 5789545 | 9184623 | 5291221 | 11686443 | 8208768 |  |  |  |
| **4^th^ run** | Sample-D1 | Sample-D2 | Sample-D3 | Sample-D4 | Sample-D5 | Sample-D6 | Sample-D7 | Sample-D8 | Sample-D9 | Sample-D10 | Sample-D11 | Sample-D12 | Sample-D13 | Sample-D14 |  |
| **Percentage mapping** | 100 | 100 | 100 | 100 | 100 | 100 | 100 | 100 | 100 | 100 | 100 | 100 | 100 | 100 |  |
| **Number of mapped reads** | 9735252 | 13256282 | 10089342 | 8123382 | 8722704 | 9136405 | 10456375 | 6249290 | 8649681 | 8399306 | 5888244 | 8612410 | 9182029 | 7617419 |  |
| **5^th^ run** | Sample-E1 | Sample-E2 | Sample-E3 | Sample-E4 | Sample-E5 | Sample-E6 | Sample-E7 | Sample-E8 | Sample-E9 | Sample-E10 | Sample-E11 | Sample-E12 | Sample-E13 | Sample-E14 | Sample-E15 |
| **Percentage mapping** | 100 | 100 | 100 | 100 | 100 | 100 | 100 | 100 | 100 | 100 | 100 | 100 | 100 | 100 | 100 |
| **Number of mapped reads** | 8355087 | 3537276 | 3719292 | 3920389 | 4332157 | 3210495 | 2792415 | 6412519 | 5478386 | 5451119 | 5414688 | 4110625 | 6457295 | 5013774 | 3482255 |
| **6^th^ run** | Sample-F1 | Sample-F2 | Sample-F3 | Sample-F4 | Sample-F5 | Sample-F6 | Sample-F7 | Sample-F8 | Sample-F9 | Sample-F10 | Sample-F11 | Sample-F12 | Sample-F13 | Sample-F14 | Sample-F15 |
| **Percentage mapping** | 100 | 100 | 100 | 100 | 100 | 100 | 100 | 100 | 100 | 100 | 100 | 100 | 100 | 100 | 100 |
| **Number of mapped reads** | 2996608 | 3117059 | 4479996 | 7700783 | 6649569 | 4999152 | 5104517 | 3879825 | 2942701 | 3207100 | 2797526 | 2630525 | 2844577 | 3013090 | 3682396 |

**Table S18. Mapping statistics of the six runs of small RNA-Sequencing generated. The percentage mapping and number of QC-passed reads that mapped against the reference genome are presented in the table for each sequenced sample.**
