# Supplementary material for: Circulating small RNA signatures differentiate accurately the subtypes of muscular dystrophies: small-RNA next-generation sequencing analytics and functional insights
Source: RNA Biol. 2022 Apr 7;19(1):507–18. doi: 10.1080/15476286.2022.2058817 (PMC8993092; doi:10.1080/15476286.2022.2058817)
Supplement: Supplemental Material [file KRNB_A_2058817_SM6377.zip › Supplementary Table S2.docx]

**Table S2. Top 20 differentially expressed miRNAs sorted by p-value for DMD.**

| **miRNA** | **logFC** | **logCPM** | **F** | **P-Value** | **FDR** | **abslogFC** |
| --- | --- | --- | --- | --- | --- | --- |
| **hsa-miR-206** | 4.769 | 11.274 | 49.953 | 2.01E-10 | 1.64E-07 | 4.769 |
| **hsa-miR-133a** | 3.295 | 6.569 | 17.785 | 5.37E-05 | 1.47E-02 | 3.295 |
| **hsa-miR-1** | 2.697 | 8.180 | 17.512 | 6.06E-05 | 1.47E-02 | 2.697 |
| **hsa-miR-514a-3p** | -6.444 | 2.584 | 17.246 | 7.17E-05 | 1.47E-02 | 6.444 |
| **hsa-miR-193b-5p** | 3.054 | 5.010 | 15.450 | 1.55E-04 | 2.53E-02 | 3.054 |
| hsa-miR-208b | 4.487 | 3.427 | 13.243 | 4.31E-04 | 5.89E-02 | 4.487 |
| hsa-miR-203 | -2.263 | 6.401 | 12.248 | 6.92E-04 | 7.09E-02 | 2.263 |
| hsa-miR-3545-5p | -2.263 | 6.401 | 12.248 | 6.92E-04 | 7.09E-02 | 2.263 |
| hsa-miR-511 | -4.931 | 1.778 | 11.453 | 1.04E-03 | 9.46E-02 | 4.931 |
| hsa-miR-877-3p | -4.474 | 1.607 | 10.985 | 1.30E-03 | 1.07E-01 | 4.474 |
| hsa-miR-15a-3p | 5.265 | 2.046 | 10.211 | 1.90E-03 | 1.41E-01 | 5.265 |
| hsa-miR-4673 | -3.859 | 1.359 | 9.759 | 2.37E-03 | 1.47E-01 | 3.859 |
| hsa-miR-4781-5p | -3.744 | 1.353 | 9.482 | 2.71E-03 | 1.47E-01 | 3.744 |
| hsa-miR-3610 | -3.688 | 1.317 | 9.442 | 2.77E-03 | 1.47E-01 | 3.688 |
| hsa-miR-1280 | -3.424 | 1.266 | 9.216 | 3.10E-03 | 1.47E-01 | 3.424 |
| hsa-miR-4773 | -3.446 | 1.316 | 9.170 | 3.17E-03 | 1.47E-01 | 3.446 |
| hsa-miR-188-5p | -3.575 | 1.321 | 9.153 | 3.19E-03 | 1.47E-01 | 3.575 |
| hsa-miR-5100 | -3.366 | 1.254 | 8.780 | 3.85E-03 | 1.47E-01 | 3.366 |
| hsa-miR-181b-3p | -3.325 | 1.261 | 8.756 | 3.90E-03 | 1.47E-01 | 3.325 |
| hsa-miR-1262 | -3.393 | 1.251 | 8.576 | 4.26E-03 | 1.47E-01 | 3.393 |
