# Supplementary material for: Circulating small RNA signatures differentiate accurately the subtypes of muscular dystrophies: small-RNA next-generation sequencing analytics and functional insights
Source: RNA Biol. 2022 Apr 7;19(1):507–18. doi: 10.1080/15476286.2022.2058817 (PMC8993092; doi:10.1080/15476286.2022.2058817)
Supplement: Supplemental Material [file KRNB_A_2058817_SM6377.zip › Supplementary Table S3.docx]

**Table S3. Top 20 differentially expressed miRNAs sorted by p-value for DM1.**

| **miRNA** | **logFC** | **logCPM** | **F** | **P-Value** | **FDR** | **abslogFC** |
| --- | --- | --- | --- | --- | --- | --- |
| **hsa-miR-4418** | -6.480 | 4.276 | 29.842 | 4.77E-08 | 4.52E-05 | 6.480 |
| **hsa-miR-142-3p** | 2.632 | 12.743 | 28.065 | 1.19E-07 | 5.64E-05 | 2.632 |
| **hsa-miR-224-5p** | 2.125 | 7.624 | 14.011 | 1.82E-04 | 4.62E-02 | 2.125 |
| **hsa-miR-199a-5p** | 2.524 | 6.232 | 13.886 | 1.95E-04 | 4.62E-02 | 2.524 |
| hsa-miR-433 | -4.337 | 3.150 | 11.842 | 5.81E-04 | 6.50E-02 | 4.337 |
| hsa-miR-3198 | -4.290 | 3.171 | 11.781 | 6.00E-04 | 6.50E-02 | 4.290 |
| hsa-miR-664-3p | 4.944 | 3.595 | 11.751 | 6.10E-04 | 6.50E-02 | 4.944 |
| hsa-miR-607 | -4.192 | 3.091 | 11.481 | 7.05E-04 | 6.50E-02 | 4.192 |
| hsa-miR-31-5p | -4.098 | 3.033 | 11.345 | 7.59E-04 | 6.50E-02 | 4.098 |
| hsa-miR-377-3p | -4.036 | 3.028 | 11.259 | 7.94E-04 | 6.50E-02 | 4.036 |
| hsa-miR-429 | -3.955 | 3.017 | 11.147 | 8.44E-04 | 6.50E-02 | 3.955 |
| hsa-miR-5697 | -3.855 | 3.010 | 11.019 | 9.04E-04 | 6.50E-02 | 3.855 |
| hsa-miR-223-3p | 1.556 | 13.421 | 10.819 | 1.01E-03 | 6.50E-02 | 1.556 |
| hsa-miR-3529-5p | -3.605 | 2.921 | 10.701 | 1.07E-03 | 6.50E-02 | 3.605 |
| hsa-miR-3691-5p | -3.514 | 2.918 | 10.663 | 1.10E-03 | 6.50E-02 | 3.514 |
| hsa-miR-631 | -3.422 | 2.884 | 10.457 | 1.22E-03 | 6.50E-02 | 3.422 |
| hsa-miR-1185-1-3p | -3.306 | 2.871 | 10.429 | 1.24E-03 | 6.50E-02 | 3.306 |
| hsa-miR-7-2-3p | -3.380 | 2.871 | 10.361 | 1.29E-03 | 6.50E-02 | 3.380 |
| hsa-miR-370 | 2.346 | 5.841 | 10.343 | 1.30E-03 | 6.50E-02 | 2.346 |
| hsa-miR-1226-5p | -3.101 | 2.828 | 10.124 | 1.47E-03 | 6.71E-02 | 3.101 |
