# Supplementary material for: Circulating small RNA signatures differentiate accurately the subtypes of muscular dystrophies: small-RNA next-generation sequencing analytics and functional insights
Source: RNA Biol. 2022 Apr 7;19(1):507–18. doi: 10.1080/15476286.2022.2058817 (PMC8993092; doi:10.1080/15476286.2022.2058817)
Supplement: Supplemental Material [file KRNB_A_2058817_SM6377.zip › Supplementary Table S4.docx]

**Table S4. Top 20 differentially expressed miRNAs sorted by p-value for DM2.**

| **miRNA** | **logFC** | **logCPM** | **F** | **P-Value** | **FDR** | **abslogFC** |
| --- | --- | --- | --- | --- | --- | --- |
| **hsa-miR-25-3p** | -1.508 | 12.396 | 16.976 | 3.81E-05 | 3.86E-02 | 1.508 |
| **hsa-miR-210** | -5.044 | 4.076 | 15.455 | 8.49E-05 | 4.30E-02 | 5.044 |
| **hsa-miR-30e-5p** | -1.604 | 9.821 | 14.478 | 1.42E-04 | 4.81E-02 | 1.604 |
| hsa-miR-363-3p | -1.922 | 7.376 | 13.046 | 3.05E-04 | 7.73E-02 | 1.922 |
| hsa-miR-3200-3p | -5.517 | 3.259 | 12.629 | 3.81E-04 | 7.73E-02 | 5.517 |
| hsa-miR-22-5p | -5.277 | 3.089 | 11.127 | 8.53E-04 | 9.22E-02 | 5.277 |
| hsa-miR-106b-3p | -1.539 | 8.478 | 11.013 | 9.07E-04 | 9.22E-02 | 1.539 |
| hsa-miR-576-5p | -2.091 | 6.012 | 10.289 | 1.34E-03 | 9.22E-02 | 2.091 |
| hsa-miR-5003-3p | 4.417 | 2.608 | 10.271 | 1.35E-03 | 9.22E-02 | 4.417 |
| hsa-miR-642a-3p | -5.071 | 2.987 | 10.128 | 1.46E-03 | 9.22E-02 | 5.071 |
| hsa-miR-642b-5p | -5.071 | 2.987 | 10.128 | 1.46E-03 | 9.22E-02 | 5.071 |
| hsa-miR-1291 | 4.184 | 2.430 | 9.801 | 1.75E-03 | 9.22E-02 | 4.184 |
| hsa-miR-4426 | 4.097 | 2.481 | 9.597 | 1.95E-03 | 9.22E-02 | 4.097 |
| hsa-miR-425-5p | -1.231 | 10.578 | 9.582 | 1.97E-03 | 9.22E-02 | 1.231 |
| hsa-miR-362-3p | 3.960 | 2.428 | 9.447 | 2.12E-03 | 9.22E-02 | 3.960 |
| hsa-miR-548i | 3.920 | 2.396 | 9.427 | 2.14E-03 | 9.22E-02 | 3.920 |
| hsa-miR-142-5p | -1.237 | 10.191 | 9.280 | 2.32E-03 | 9.22E-02 | 1.237 |
| hsa-miR-4685-5p | 3.570 | 2.294 | 9.163 | 2.47E-03 | 9.22E-02 | 3.570 |
| hsa-miR-203 | 1.550 | 7.593 | 9.137 | 2.51E-03 | 9.22E-02 | 1.550 |
| hsa-miR-3545-5p | 1.550 | 7.593 | 9.137 | 2.51E-03 | 9.22E-02 | 1.550 |
