# Supplementary material for: Circulating small RNA signatures differentiate accurately the subtypes of muscular dystrophies: small-RNA next-generation sequencing analytics and functional insights
Source: RNA Biol. 2022 Apr 7;19(1):507–18. doi: 10.1080/15476286.2022.2058817 (PMC8993092; doi:10.1080/15476286.2022.2058817)
Supplement: Supplemental Material [file KRNB_A_2058817_SM6377.zip › Supplementary Table S5.docx]

**Table S5. Top 20 differentially expressed miRNAs sorted by p-value for FSHD1.**

| **miRNA** | **logFC** | **logCPM** | **F** | **P-Value** | **FDR** | **abslogFC** |
| --- | --- | --- | --- | --- | --- | --- |
| **hsa-miR-206** | 2.432766 | 8.765409 | 26.7711 | 2.32E-07 | 0.000216 | hsa-miR-206 |
| **hsa-miR-223-3p** | 1.71112 | 12.94393 | 20.58728 | 5.75E-06 | 0.002676 | hsa-miR-223-3p |
| **hsa-miR-199b-5p** | 3.548217 | 4.438202 | 16.37994 | 5.21E-05 | 0.016178 | hsa-miR-199b-5p |
| hsa-miR-4755-3p | -4.96382 | 2.802242 | 13.35101 | 0.000259 | 0.060351 | hsa-miR-4755-3p |
| hsa-miR-3193 | -4.63787 | 2.707256 | 12.02526 | 0.000527 | 0.070261 | hsa-miR-3193 |
| hsa-miR-499a-5p | 5.169973 | 2.90274 | 12.0191 | 0.000528 | 0.070261 | hsa-miR-499a-5p |
| hsa-miR-499b-3p | 5.169973 | 2.90274 | 12.0191 | 0.000528 | 0.070261 | hsa-miR-499b-3p |
| hsa-miR-4683 | -4.56932 | 2.668664 | 11.67045 | 0.000637 | 0.074124 | hsa-miR-4683 |
| hsa-miR-1277-5p | -3.62419 | 3.681674 | 11.21669 | 0.000813 | 0.075979 | hsa-miR-1277-5p |
| hsa-miR-127-3p | 2.267318 | 5.288602 | 11.20951 | 0.000816 | 0.075979 | hsa-miR-127-3p |
| hsa-let-7a-3p | 4.865136 | 2.807887 | 10.83593 | 0.000998 | 0.084479 | hsa-let-7a-3p |
| hsa-miR-2355-3p | -3.88079 | 2.391798 | 9.738807 | 0.001808 | 0.119624 | hsa-miR-2355-3p |
| hsa-miR-5697 | -3.87738 | 2.38465 | 9.724377 | 0.001822 | 0.119624 | hsa-miR-5697 |
| hsa-miR-337-5p | -3.82413 | 2.363828 | 9.609061 | 0.00194 | 0.119624 | hsa-miR-337-5p |
| hsa-miR-449a | -3.73839 | 2.320349 | 9.396714 | 0.002178 | 0.119624 | hsa-miR-449a |
| hsa-miR-627 | -3.48642 | 2.257812 | 9.07189 | 0.002601 | 0.119624 | hsa-miR-627 |
| hsa-miR-3127-3p | -3.358 | 2.274587 | 8.858247 | 0.002923 | 0.119624 | hsa-miR-3127-3p |
| hsa-miR-186-3p | -3.27764 | 2.233208 | 8.814059 | 0.002995 | 0.119624 | hsa-miR-186-3p |
| hsa-miR-877-5p | 4.285413 | 2.611948 | 8.784936 | 0.003043 | 0.119624 | hsa-miR-877-5p |
| hsa-miR-1238 | -3.19644 | 2.209205 | 8.706231 | 0.003177 | 0.119624 | hsa-miR-1238 |
