# Supplementary material for: Circulating small RNA signatures differentiate accurately the subtypes of muscular dystrophies: small-RNA next-generation sequencing analytics and functional insights
Source: RNA Biol. 2022 Apr 7;19(1):507–18. doi: 10.1080/15476286.2022.2058817 (PMC8993092; doi:10.1080/15476286.2022.2058817)
Supplement: Supplemental Material [file KRNB_A_2058817_SM6377.zip › Supplementary Table S6.docx]

**Table S6. Top 20 differentially expressed miRNAs sorted by p-value for LGMD R1 calpain3-related.**

| **miRNA** | **logFC** | **logCPM** | **F** | **P-Value** | **FDR** | **abslogFC** |
| --- | --- | --- | --- | --- | --- | --- |
| **hsa-miR-142-3p** | 2.680 | 12.364 | 35.934 | 2.10E-09 | 1.92E-06 | 2.680 |
| **hsa-miR-223-3p** | *2.324* | *14.122* | *31.330* | *2.22E-08* | *6.99E-06* | *2.324* |
| **hsa-miR-143-3p** | 2.639 | 10.743 | 31.270 | 2.29E-08 | 6.99E-06 | 2.639 |
| **hsa-miR-486-5p** | *-1.900* | *16.624* | *24.913* | *6.08E-07* | *1.39E-04* | *1.900* |
| **hsa-miR-208b** | 6.626 | 4.164 | 23.572 | 1.22E-06 | 2.23E-04 | 6.626 |
| **hsa-miR-486-3p** | *-1.826* | *16.348* | *22.769* | *1.85E-06* | *2.82E-04* | *1.826* |
| **hsa-miR-4418** | -6.384 | 3.736 | 19.632 | 9.47E-06 | 1.24E-03 | 6.384 |
| **hsa-miR-1** | *2.303* | *7.703* | *16.266* | *5.54E-05* | *6.33E-03* | *2.303* |
| **hsa-miR-3613-5p** | 2.542 | 6.671 | 15.304 | 9.20E-05 | 9.35E-03 | 2.542 |
| **hsa-miR-199a-3p** | *1.634* | *12.089* | *14.133* | *1.71E-04* | *1.57E-02* | *1.634* |
| **hsa-miR-140-5p** | 2.204 | 7.179 | 13.395 | 2.53E-04 | 1.88E-02 | 2.204 |
| **hsa-miR-3074-5p** | *1.804* | *9.157* | *13.173* | *2.85E-04* | *1.88E-02* | *1.804* |
| **hsa-miR-24-3p** | 1.685 | 10.245 | 13.120 | 2.93E-04 | 1.88E-02 | 1.685 |
| **hsa-miR-450b-5p** | *5.430* | *3.343* | *13.004* | *3.12E-04* | *1.88E-02* | *5.430* |
| **hsa-miR-199b-3p** | 1.615 | 11.089 | 12.916 | 3.27E-04 | 1.88E-02 | 1.615 |
| **hsa-miR-4732-5p** | *-1.966* | *7.908* | *12.808* | *3.46E-04* | *1.88E-02* | *1.966* |
| **hsa-miR-499a-5p** | 5.409 | 3.304 | 12.689 | 3.69E-04 | 1.88E-02 | 5.409 |
| **hsa-miR-499b-3p** | *5.409* | *3.304* | *12.689* | *3.69E-04* | *1.88E-02* | *5.409* |
| **hsa-miR-337-5p** | 5.262 | 3.234 | 11.981 | 5.39E-04 | 2.60E-02 | 5.262 |
| **hsa-miR-582-5p** | *5.154* | *3.157* | *11.434* | *7.23E-04* | *3.31E-02* | *5.154* |
