# Supplementary material for: Circulating small RNA signatures differentiate accurately the subtypes of muscular dystrophies: small-RNA next-generation sequencing analytics and functional insights
Source: RNA Biol. 2022 Apr 7;19(1):507–18. doi: 10.1080/15476286.2022.2058817 (PMC8993092; doi:10.1080/15476286.2022.2058817)
Supplement: Supplemental Material [file KRNB_A_2058817_SM6377.zip › Supplementary Table S7.docx]

**Table S7. Summary of PCA components.**

| **Importance of components:** |  |  |  |  |  |  |  |  |  |
| --- | --- | --- | --- | --- | --- | --- | --- | --- | --- |
| **DMD** | **PC1** | **PC2** | **PC3** | **PC4** | **PC5** | **PC6** | **PC7** | **PC8** |  |
| Standard deviation | 3.4588 | 1.15143 | 0.92094 | 0.71909 | 0.61008 | 0.52125 | 0.44936 | 0.39674 |  |
| Proportion of Variance | 0.7477 | 0.08286 | 0.05301 | 0.03232 | 0.02326 | 0.01698 | 0.01262 | 0.00984 |  |
| Cumulative Proportion | 0.7477 | 0.83056 | 0.88357 | 0.91589 | 0.93915 | 0.95613 | 0.96875 | 0.97859 |  |
| **DMD** | **PC9** | **PC10** | **PC11** | **PC12** | **PC13** | **PC14** | **PC15** | **PC16** |  |
| Standard deviation | 0.33817 | 0.26729 | 0.25063 | 0.21029 | 0.15401 | 0.128 | 0.0809 | 0.0551 |  |
| Proportion of Variance | 0.00715 | 0.00447 | 0.00393 | 0.00276 | 0.00148 | 0.00102 | 0.00041 | 0.00019 |  |
| Cumulative Proportion | 0.98574 | 0.9902 | 0.99413 | 0.99689 | 0.99838 | 0.9994 | 0.99981 | 1 |  |
|  |  |  |  |  |  |  |  |  |  |
| **DM1** | **PC1** | **PC2** | **PC3** | **PC4** | **PC5** | **PC6** | **PC7** | **PC8** |  |
| Standard deviation | 3.9045 | 0.76951 | 0.53808 | 0.40439 | 0.39055 | 0.3594 | 0.33032 | 0.3 |  |
| Proportion of Variance | 0.8968 | 0.03483 | 0.01703 | 0.00962 | 0.00897 | 0.0076 | 0.00642 | 0.0053 |  |
| Cumulative Proportion | 0.8968 | 0.9316 | 0.94863 | 0.95825 | 0.96722 | 0.9748 | 0.98124 | 0.9865 |  |
| **DM1** | **PC9** | **PC10** | **PC11** | **PC12** | **PC13** | **PC14** | **PC15** | **PC16** | **PC17** |
| Standard deviation | 0.25459 | 0.22859 | 0.18815 | 0.17195 | 0.14114 | 0.1271 | 0.07658 | 0.05689 | 0.04171 |
| Proportion of Variance | 0.00381 | 0.00307 | 0.00208 | 0.00174 | 0.00117 | 0.00095 | 0.00034 | 0.00019 | 0.0001 |
| Cumulative Proportion | 0.99035 | 0.99342 | 0.9955 | 0.99724 | 0.99841 | 0.99936 | 0.99971 | 0.9999 | 1 |
|  |  |  |  |  |  |  |  |  |  |
| **DM2** | **PC1** | **PC2** | **PC3** | **PC4** | **PC5** | **PC6** | **PC7** | **PC8** |  |
| Standard deviation | 3.7872 | 0.89645 | 0.44015 | 0.40352 | 0.33636 | 0.3019 | 0.25818 | 0.23055 |  |
| Proportion of Variance | 0.8964 | 0.05023 | 0.01211 | 0.01018 | 0.00707 | 0.0057 | 0.00417 | 0.00332 |  |
| Cumulative Proportion | 0.8964 | 0.94665 | 0.95876 | 0.96894 | 0.97601 | 0.9817 | 0.98587 | 0.98919 |  |
| **DM2** | **PC9** | **PC10** | **PC11** | **PC12** | **PC13** | **PC14** | **PC15** | **PC16** |  |
| Standard deviation | 0.20893 | 0.20249 | 0.16332 | 0.13896 | 0.12955 | 0.1058 | 0.09194 | 0.07632 |  |
| Proportion of Variance | 0.00273 | 0.00256 | 0.00167 | 0.00121 | 0.00105 | 0.0007 | 0.00053 | 0.00036 |  |
| Cumulative Proportion | 0.99192 | 0.99448 | 0.99615 | 0.99736 | 0.99841 | 0.9991 | 0.99964 | 1 |  |
|  |  |  |  |  |  |  |  |  |  |
| **FSHD1** | **PC1** | **PC2** | **PC3** | **PC4** | **PC5** | **PC6** | **PC7** | **PC8** |  |
| Standard deviation | 3.6659 | 0.9148 | 0.71656 | 0.5337 | 0.44865 | 0.41833 | 0.36523 | 0.32584 |  |
| Proportion of Variance | 0.8399 | 0.0523 | 0.03209 | 0.0178 | 0.01258 | 0.01094 | 0.00834 | 0.00664 |  |
| Cumulative Proportion | 0.8399 | 0.8922 | 0.92432 | 0.9421 | 0.9547 | 0.96564 | 0.97398 | 0.98061 |  |
| **FSHD1** | **PC9** | **PC10** | **PC11** | **PC12** | **PC13** | **PC14** | **PC15** | **PC16** |  |
| Standard deviation | 0.31172 | 0.25491 | 0.2275 | 0.20473 | 0.1832 | 0.10856 | 0.08232 | 0.04772 |  |
| Proportion of Variance | 0.00607 | 0.00406 | 0.00323 | 0.00262 | 0.0021 | 0.00074 | 0.00042 | 0.00014 |  |
| Cumulative Proportion | 0.98668 | 0.99075 | 0.99398 | 0.9966 | 0.9987 | 0.99943 | 0.99986 | 1 |  |
|  |  |  |  |  |  |  |  |  |  |
| **LGMD R1 calpain3-related** | **PC1** | **PC2** | **PC3** | **PC4** | **PC5** | **PC6** | **PC7** | **PC8** |  |
| Standard deviation | 3.8642 | 0.80353 | 0.31074 | 0.29896 | 0.24392 | 0.20564 | 0.17953 | 0.1579 |  |
| Proportion of Variance | 0.9332 | 0.04035 | 0.00604 | 0.00559 | 0.00372 | 0.00264 | 0.00201 | 0.00156 |  |
| Cumulative Proportion | 0.9332 | 0.97358 | 0.97962 | 0.98521 | 0.98892 | 0.99157 | 0.99358 | 0.99514 |  |
| **LGMD R1 calpain3-related** | **PC9** | **PC10** | **PC11** | **PC12** | **PC13** | **PC14** | **PC15** | **PC16** |  |
| Standard deviation | 0.14135 | 0.13086 | 0.11013 | 0.10765 | 0.08323 | 0.06741 | 0.06369 | 0.03758 |  |
| Proportion of Variance | 0.00125 | 0.00107 | 0.00076 | 0.00072 | 0.00043 | 0.00028 | 0.00025 | 0.00009 |  |
| Cumulative Proportion | 0.99639 | 0.99746 | 0.99822 | 0.99894 | 0.99937 | 0.99966 | 0.99991 | 1 |  |
