# Supplementary material for: Circulating small RNA signatures differentiate accurately the subtypes of muscular dystrophies: small-RNA next-generation sequencing analytics and functional insights
Source: RNA Biol. 2022 Apr 7;19(1):507–18. doi: 10.1080/15476286.2022.2058817 (PMC8993092; doi:10.1080/15476286.2022.2058817)
Supplement: Supplemental Material [file KRNB_A_2058817_SM6377.zip › Supplementary Table S8.docx]

**Table S8. DMD LOOCV panel of pooled top scoring miRNAs.**

|  | **logFC** | **logCPM** | **F** | **P-Value** | **FDR** |
| --- | --- | --- | --- | --- | --- |
| hsa-miR-1 | 2.660626 | 8.218111 | 17.43848 | 0.000131 | 0.025321 |
| hsa-miR-133a | 3.180964 | 6.544931 | 17.86232 | 0.000168 | 0.029383 |
| hsa-miR-15a-3p | 5.438486 | 2.104742 | 10.55267 | 0.001541 | 0.174367 |
| hsa-miR-193b-5p | 2.966234 | 5.052072 | 14.10214 | 0.000417 | 0.050928 |
| hsa-miR-203 | -2.45081 | 6.51103 | 13.99564 | 0.000399 | 0.048721 |
| hsa-miR-206 | 4.698692 | 11.24288 | 51.17002 | 2.28E-09 | 1.81E-06 |
| hsa-miR-208b | 4.74442 | 3.499817 | 13.60329 | 0.000496 | 0.060351 |
| hsa-miR-3131 | -5.93049 | 2.432248 | 13.62507 | 0.000308 | 0.035233 |
| hsa-miR-3545-5p | -2.45496 | 6.511571 | 14.11656 | 0.000386 | 0.04848 |
| hsa-miR-511 | -5.19816 | 1.854763 | 11.79248 | 0.000934 | 0.098056 |
| hsa-miR-514a-3p | -6.313 | 2.574373 | 16.2323 | 0.00012 | 0.025971 |
| hsa-miR-877-3p | -4.57958 | 1.602242 | 11.045 | 0.001299 | 0.126525 |
