# Supplementary material for: Circulating small RNA signatures differentiate accurately the subtypes of muscular dystrophies: small-RNA next-generation sequencing analytics and functional insights
Source: RNA Biol. 2022 Apr 7;19(1):507–18. doi: 10.1080/15476286.2022.2058817 (PMC8993092; doi:10.1080/15476286.2022.2058817)
Supplement: Supplemental Material [file KRNB_A_2058817_SM6377.zip › Supplementary Table S9.docx]

**Table S9. DM1 LOOCV panel of pooled top scoring miRNAs.**

|  | **logFC** | **logCPM** | **F** | **P-Value** | **FDR** |
| --- | --- | --- | --- | --- | --- |
| hsa-miR-1277-5p | 5.680758 | 4.078305 | 14.60268 | 0.000133 | 0.024881 |
| hsa-miR-142-3p | 2.709656 | 12.79293 | 26.36106 | 6.75E-07 | 0.000341 |
| hsa-miR-199a-5p | 2.59529 | 6.290718 | 13.94204 | 0.000288 | 0.055402 |
| hsa-miR-223-3p | 1.74603 | 13.47099 | 12.40453 | 0.000588 | 0.063562 |
| hsa-miR-224-5p | 2.163463 | 7.644183 | 13.64021 | 0.000321 | 0.055345 |
| hsa-miR-31-5p | -4.20921 | 3.025286 | 10.95657 | 0.000992 | 0.084063 |
| hsa-miR-3198 | -4.40575 | 3.198649 | 11.28787 | 0.000831 | 0.086622 |
| hsa-miR-3529-5p | -3.91017 | 2.845599 | 10.90796 | 0.00096 | 0.081198 |
| hsa-miR-370 | 2.646672 | 5.971865 | 12.56895 | 0.000468 | 0.06699 |
| hsa-miR-377-3p | -3.89694 | 2.949614 | 11.04623 | 0.000891 | 0.090667 |
| hsa-miR-381 | 4.244589 | 4.621325 | 12.46277 | 0.000417 | 0.05124 |
| hsa-miR-382-3p | 4.616281 | 3.475975 | 10.28852 | 0.001342 | 0.110305 |
| hsa-miR-429 | -4.0328 | 2.987698 | 11.14978 | 0.000843 | 0.090667 |
| hsa-miR-4326 | -4.40012 | 3.345377 | 11.10373 | 0.000864 | 0.077742 |
| hsa-miR-433 | -4.25804 | 3.100712 | 11.0302 | 0.000944 | 0.082915 |
| hsa-miR-4418 | -6.37142 | 4.204043 | 26.23952 | 5.01E-06 | 0.00237 |
| hsa-miR-4446-3p | 5.344482 | 3.876429 | 12.50211 | 0.000408 | 0.047571 |
| hsa-miR-5187-5p | -5.06155 | 3.61391 | 14.76194 | 0.000123 | 0.022697 |
| hsa-miR-5697 | -4.16715 | 3.022204 | 10.99976 | 0.000951 | 0.080037 |
| hsa-miR-607 | -4.11482 | 3.079138 | 10.68285 | 0.001118 | 0.09093 |
| hsa-miR-628-5p | 5.184822 | 3.693665 | 11.63124 | 0.000651 | 0.057288 |
| hsa-miR-664-3p | 5.05284 | 3.612783 | 12.04795 | 0.000559 | 0.082116 |
| hsa-miR-885-5p | 5.210201 | 3.791189 | 11.7584 | 0.000608 | 0.062889 |
