# Supplementary figures and images for: Circulating small RNA signatures differentiate accurately the subtypes of muscular dystrophies: small-RNA next-generation sequencing analytics and functional insights
Source: RNA Biol. 2022 Apr 7;19(1):507–18. doi: 10.1080/15476286.2022.2058817 (PMC8993092; doi:10.1080/15476286.2022.2058817)

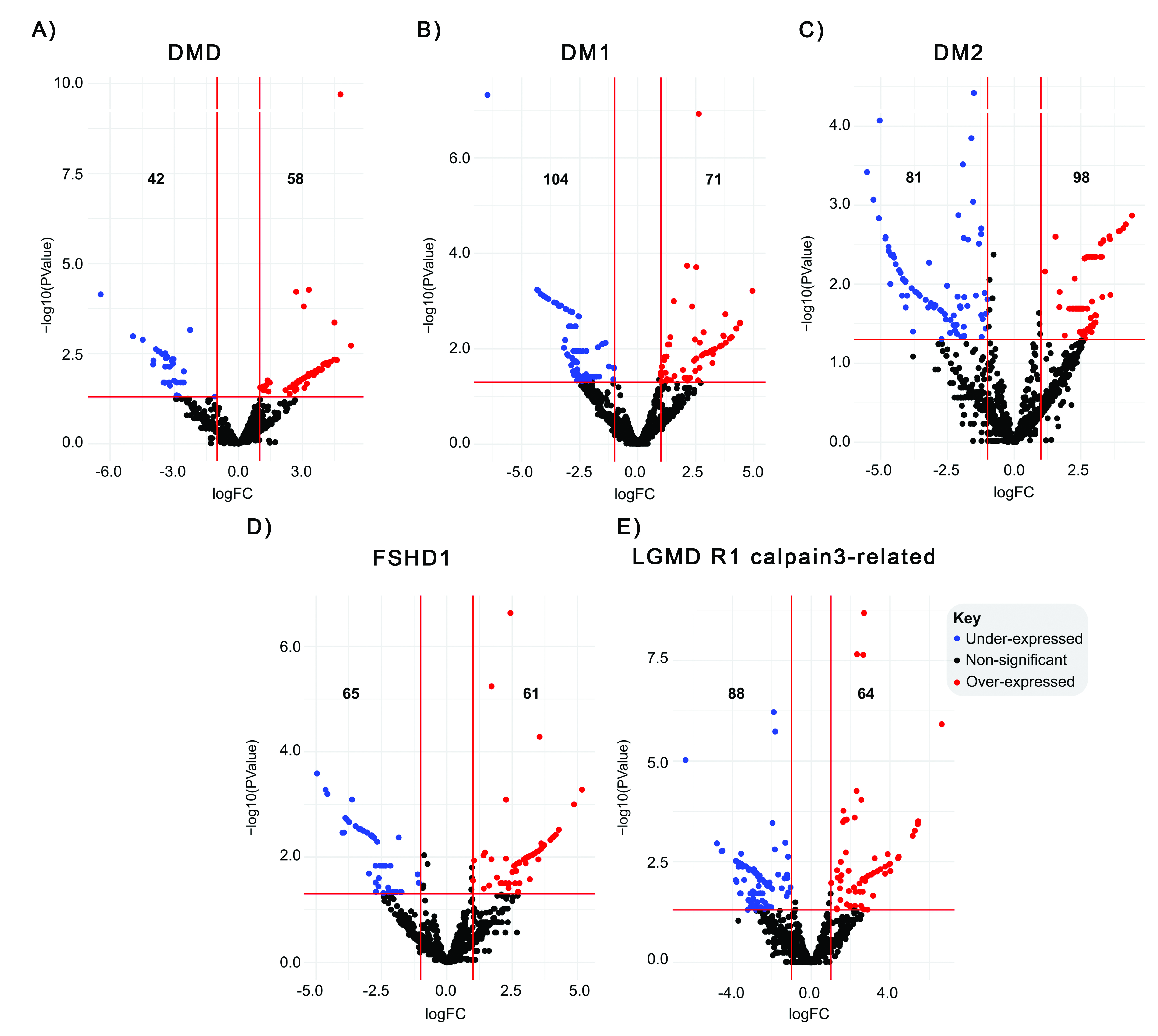

Supplement: Supplemental Material [file KRNB_A_2058817_SM6377.zip › Supplementary Figure S1.jpg]

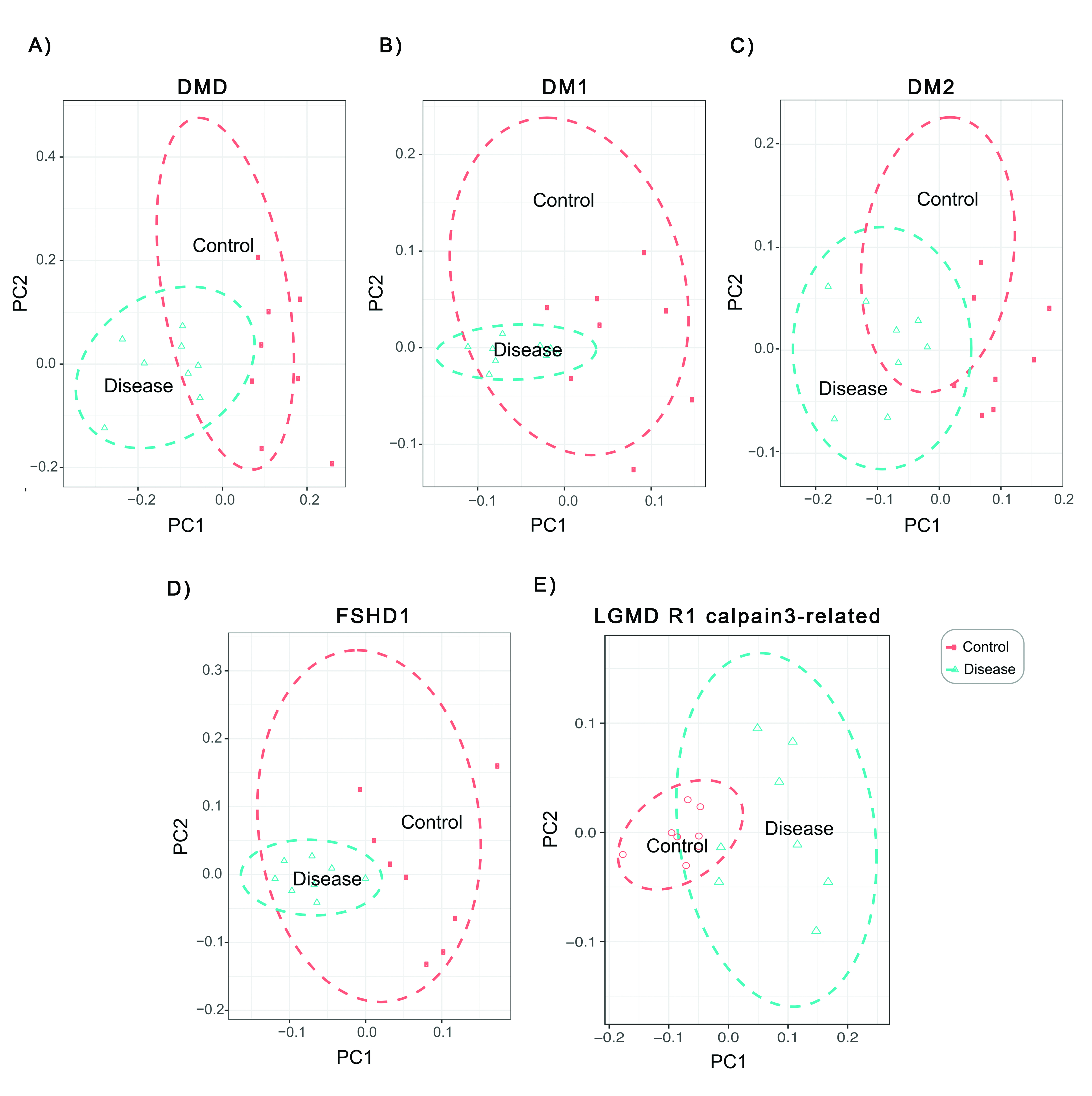

Supplement: Supplemental Material [file KRNB_A_2058817_SM6377.zip › Supplementary Figure S2.jpg]

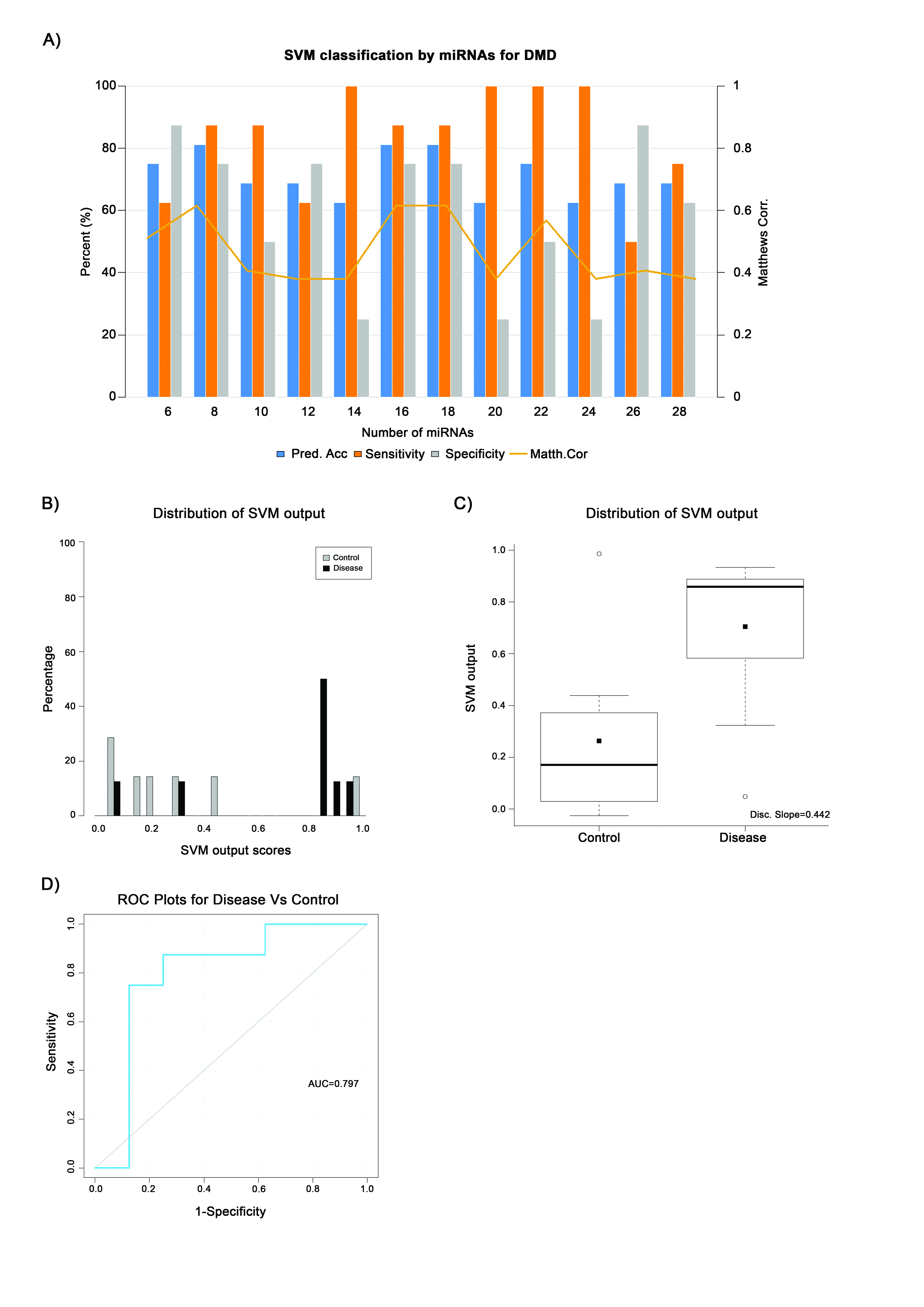

Supplement: Supplemental Material [file KRNB_A_2058817_SM6377.zip › Supplementary Figure S3.jpg]

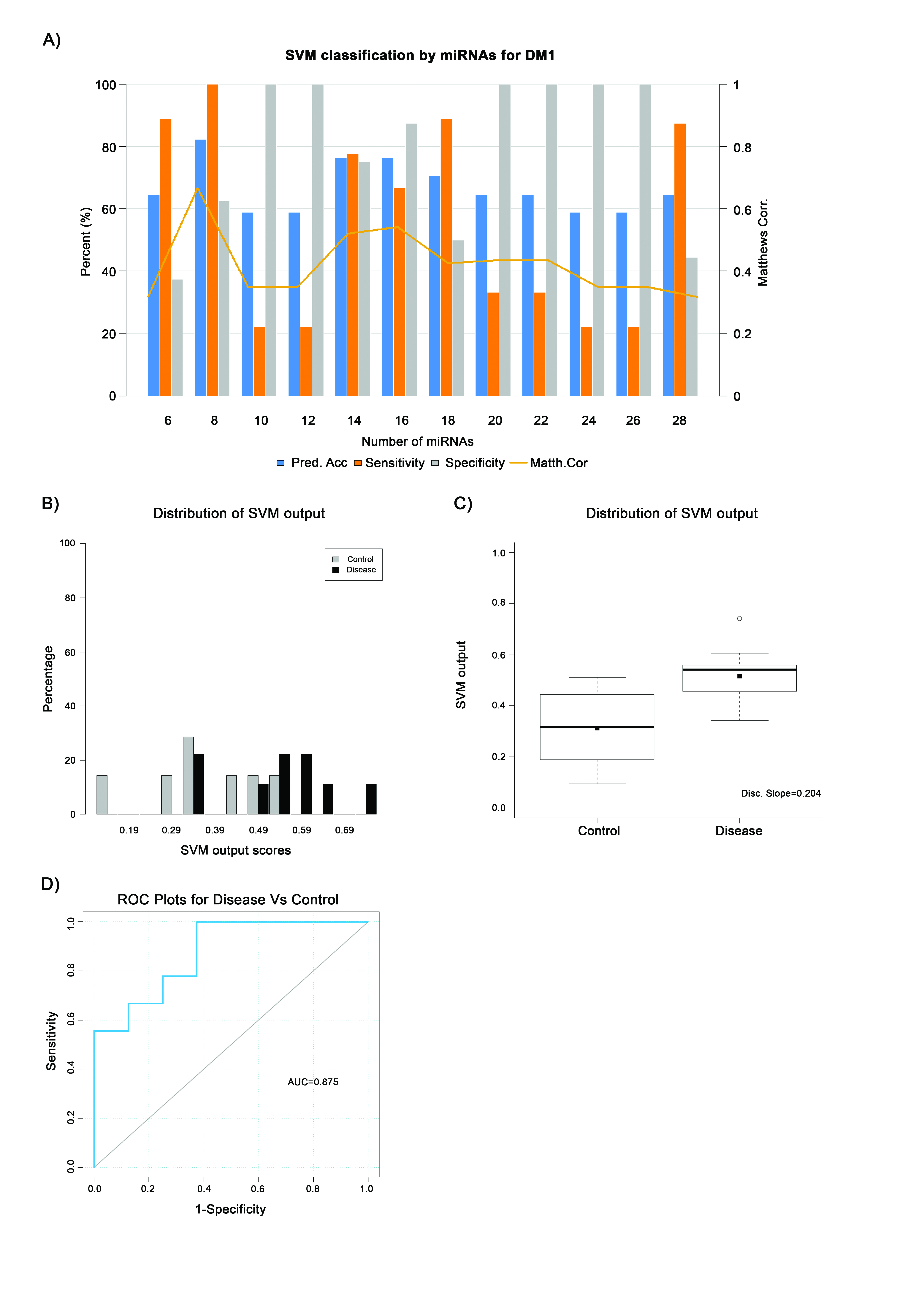

Supplement: Supplemental Material [file KRNB_A_2058817_SM6377.zip › Supplementary Figure S4.jpg]

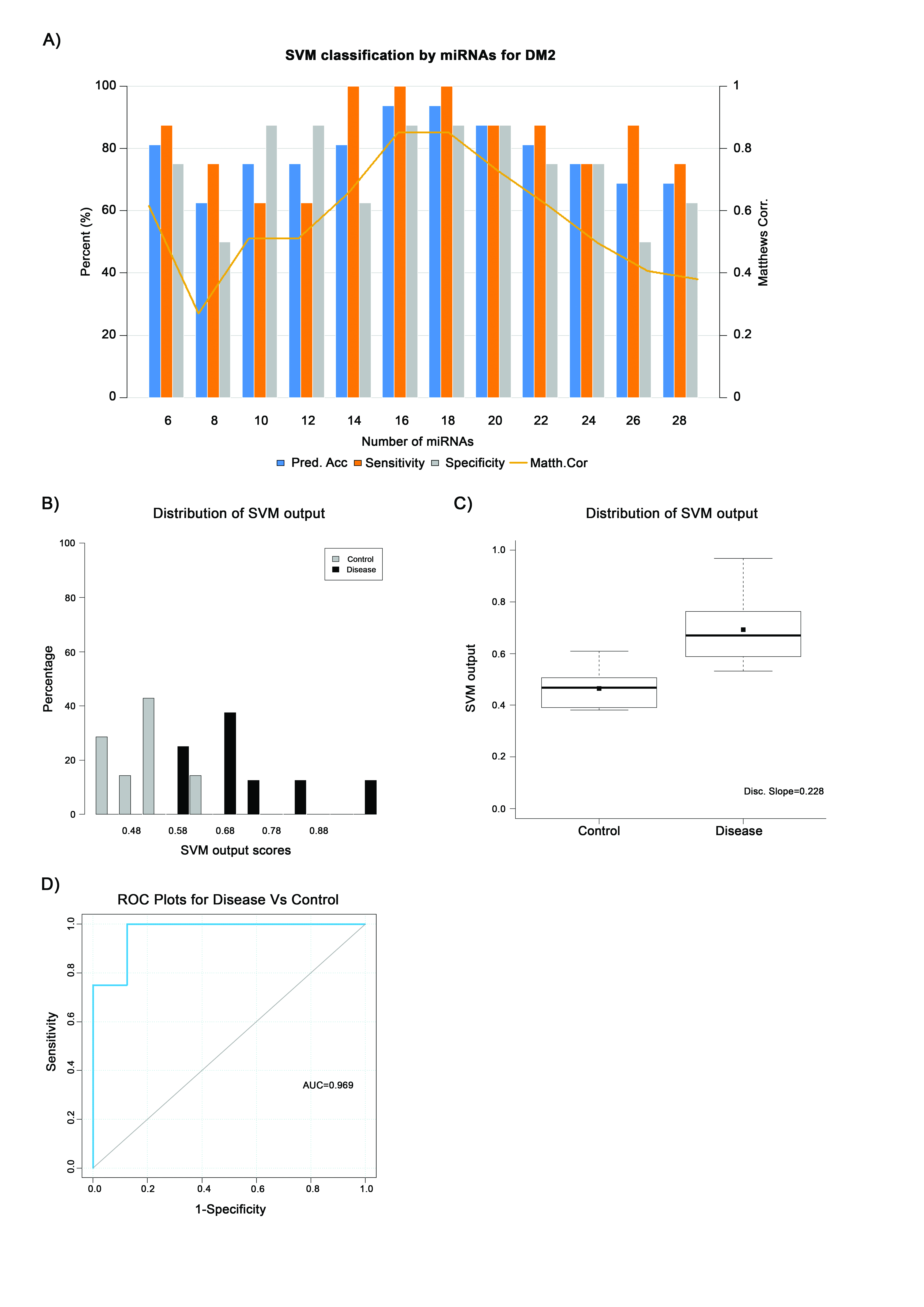

Supplement: Supplemental Material [file KRNB_A_2058817_SM6377.zip › Supplementary Figure S5.jpg]

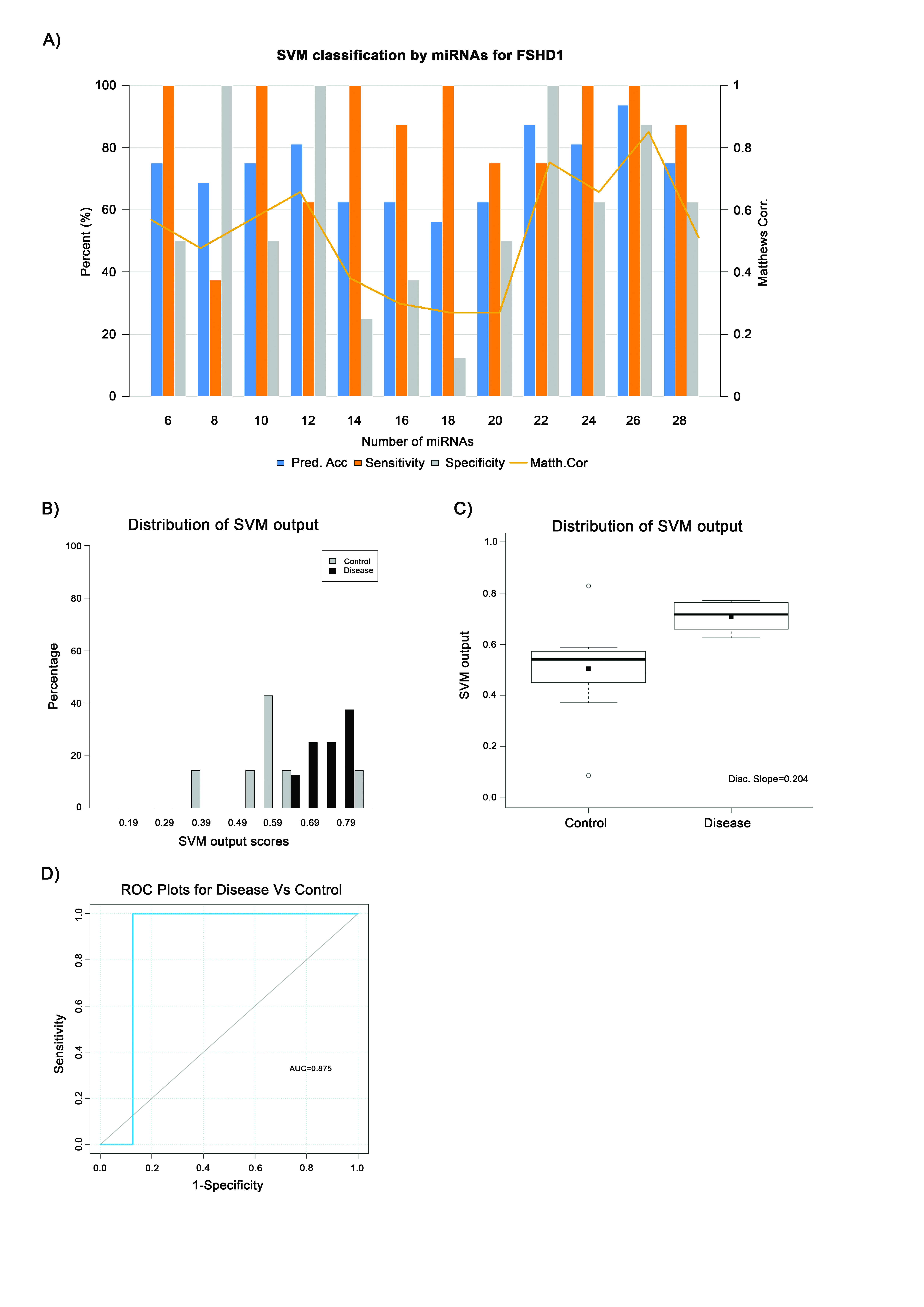

Supplement: Supplemental Material [file KRNB_A_2058817_SM6377.zip › Supplementary Figure S6.jpg]

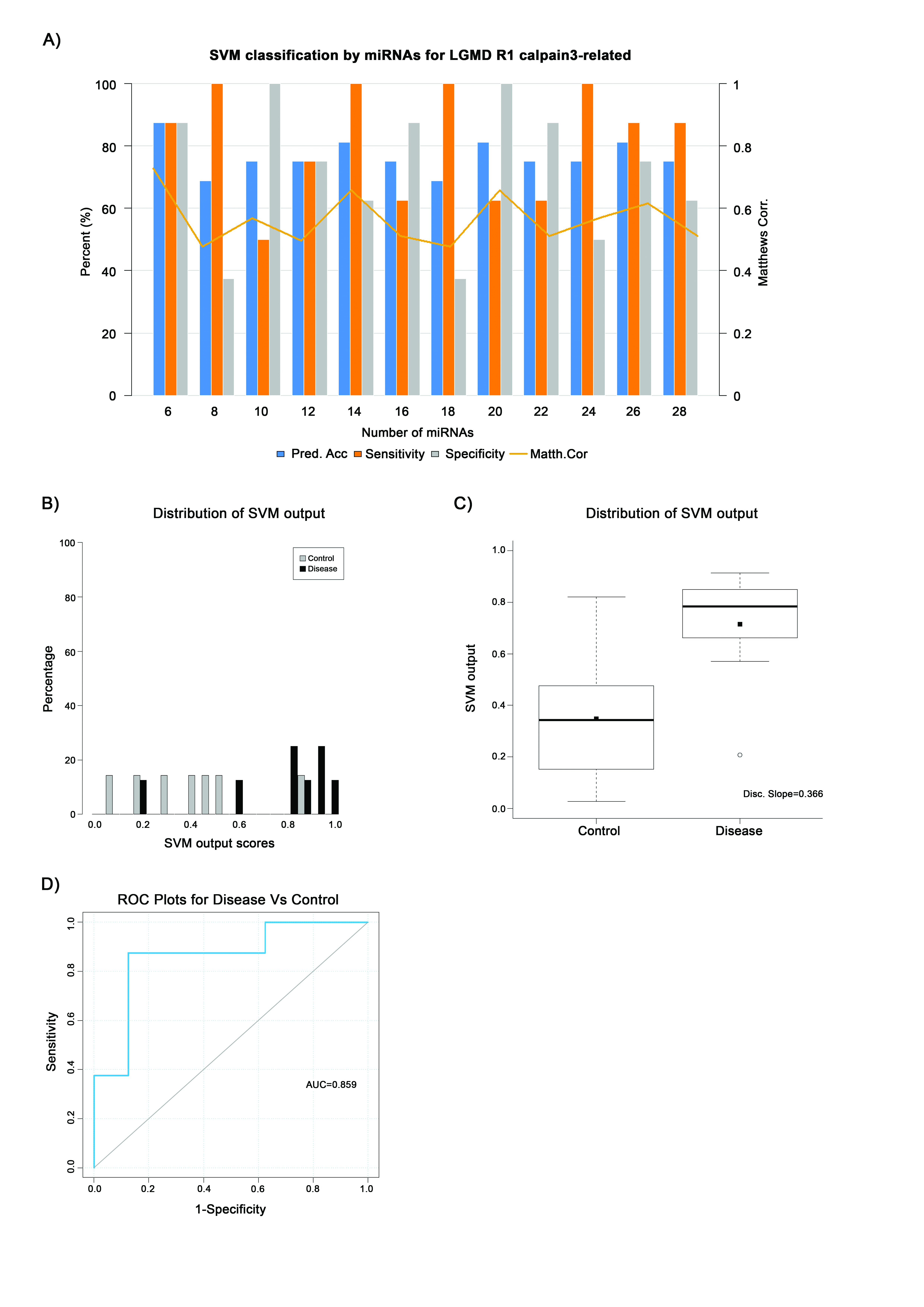

Supplement: Supplemental Material [file KRNB_A_2058817_SM6377.zip › Supplementary Figure S7.jpg]
